# Development of [^18^F]LU14 for PET Imaging of Cannabinoid Receptor Type 2 in the Brain

**DOI:** 10.3390/ijms22158051

**Published:** 2021-07-28

**Authors:** Rodrigo Teodoro, Daniel Gündel, Winnie Deuther-Conrad, Lea Ueberham, Magali Toussaint, Guy Bormans, Peter Brust, Rareş-Petru Moldovan

**Affiliations:** 1 Helmholtz-Zentrum Dresden-Rossendorf (HZDR), Institute of Radiopharmaceutical Cancer Research, Department of Neuroradiopharmaceuticals, Research Site Leipzig, 04318 Leipzig, Germany; r.teodoro@hzdr.de (R.T.); d.guendel@hzdr.de (D.G.); w.deuther-conrad@hzdr.de (W.D.-C.); uelea@web.de (L.U.); m.toussaint@hzdr.de (M.T.); p.brust@hzdr.de (P.B.); 2 Radiopharmaceutical Research, Department of Pharmaceutical and Pharmacological Sciences, KU Leuven, BE-3000 Leuven, Belgium; guy.bormans@kuleuven.be; 3 The Lübeck Institute of Experimental Dermatology, University Medical Center Schleswig-Holstein, 23562 Lübeck, Germany

**Keywords:** cannabinoid receptor type 2, naphtyrid-2-one, binding affinity, radiochemistry, fluorine-18 labelling, brain, positron emission tomography

## Abstract

Cannabinoid receptors type 2 (CB2R) represent an attractive therapeutic target for neurodegenerative diseases and cancer. Aiming at the development of a positron emission tomography (PET) radiotracer to monitor receptor density and/or occupancy during a CB2R-tailored therapy, we herein describe the radiosynthesis of *cis*-[^18^F]1-(4-fluorobutyl-*N*-((1s,4s)-4-methylcyclohexyl)-2-oxo-1,2-dihydro-1,8-naphthyridine-3-carboxamide ([^18^F]**LU14**) starting from the corresponding mesylate precursor. The first biological evaluation revealed that [^18^F]**LU14** is a highly affine CB2R radioligand with >80% intact tracer in the brain at 30 min p.i. Its further evaluation by PET in a well-established rat model of CB2R overexpression demonstrated its ability to selectively image the CB2R in the brain and its potential as a tracer to further investigate disease-related changes in CB2R expression.

## 1. Introduction

The psychoactive effect of the well-known drug marijuana is caused by the active ingredient (−)-Δ9-trans-tetrahydrocannabinol (THC), which abundantly occurs in the cannabis plant [1]. The investigation of the mechanism through which THC manifests its biological activity led to the discovery of the endocannabinoid system. It comprises a class of transmembrane proteins that belongs to the superfamily of G-protein-coupled receptors; their endogenous ligands (endocannabinoids), such as anandamide (AEA) [2] and 2-arachidonoylglycerol (2-AG); the endocannabinoid-synthesising (diacylglycerol lipase *N*-acylphosphatidyl-ethanolamine phospholipase D) and -degrading enzymes (fatty acid amide hydrolase, monoacylglycerol lipase), as well as the endocannabinoid transporters [3,4]. Two types of cannabinoid receptors, namely cannabinoid receptors type 1 (CB1R) [5] and cannabinoid receptors type 2 (CB2R) [6], were identified so far and are being thoroughly investigated [7,8]. Both CB1R and CB2R transduce signals from extracellular to intracellular space by inhibiting the activity of adenylyl cyclase over G_i/o_ proteins, thereby suppressing the subsequent cyclic adenosine monophosphate (cAMP) pathway [9,10]. While the CB1R is associated with the central nervous system and is abundantly expressed in the brain (highest density in the hippocampus, cerebellum and striatum), the CB2R is found in the spleen, tonsils and the thymus gland and modulates immune cell functions. In the brain, the CB2R are present at low levels [11,12,13,14,15], and their overexpression is associated with neurodegenerative diseases such as Alzheimer’s disease, Parkinson’s disease, Huntington’s disease and cancer [16,17,18,19,20,21,22,23,24,25,26,27,28,29,30]. More recently, it has been shown that CB2R is expressed in dopaminergic neurons in the midbrain and, in glutamatergic neurons in the red nucleus, modulates motor behaviour in mice [31]. Others have shown that the selective activation of the CB2R receptor results in cell apoptosis, the inhibition of tumor cell growth and the inhibition of neo-angiogenesis [32,33,34]. Moreover, selective CB2R agonists were successfully used to suppress inflammatory and neuropathic pain without psychotropic side effects caused by the use of THC or other CB1R-related pharmaceuticals [35,36]. The overexpression of CB2R receptors was correlated with microglial polarisation from a pro-inflammatory M1 to anti-inflammatory M2 state by a yet not fully elucidated mechanism [37]. Thus, CB2R are regarded as a highly valuable marker for the early detection and treatment of several neuropathological conditions [38,39]. The investigation of CB2R as a target to develop novel therapeutic and early diagnostic strategies gained importance over the past years. In the search for novel agonists and antagonists acting at the CB2R, several classes of compounds were thoroughly investigated by systematic SAR (structure–activity relationship) studies [40]. The development of novel potent and selective CB2R ligands is supported by computer-based docking studies and accelerated by the recently reported crystal structure of the receptor [41]. The non-invasive imaging of CB2R with positron emission tomography (PET) is of great interest, as demonstrated by the large number of positron-emitting radioligands developed over the past years [42,43,44,45,46].

**Figure 1:**
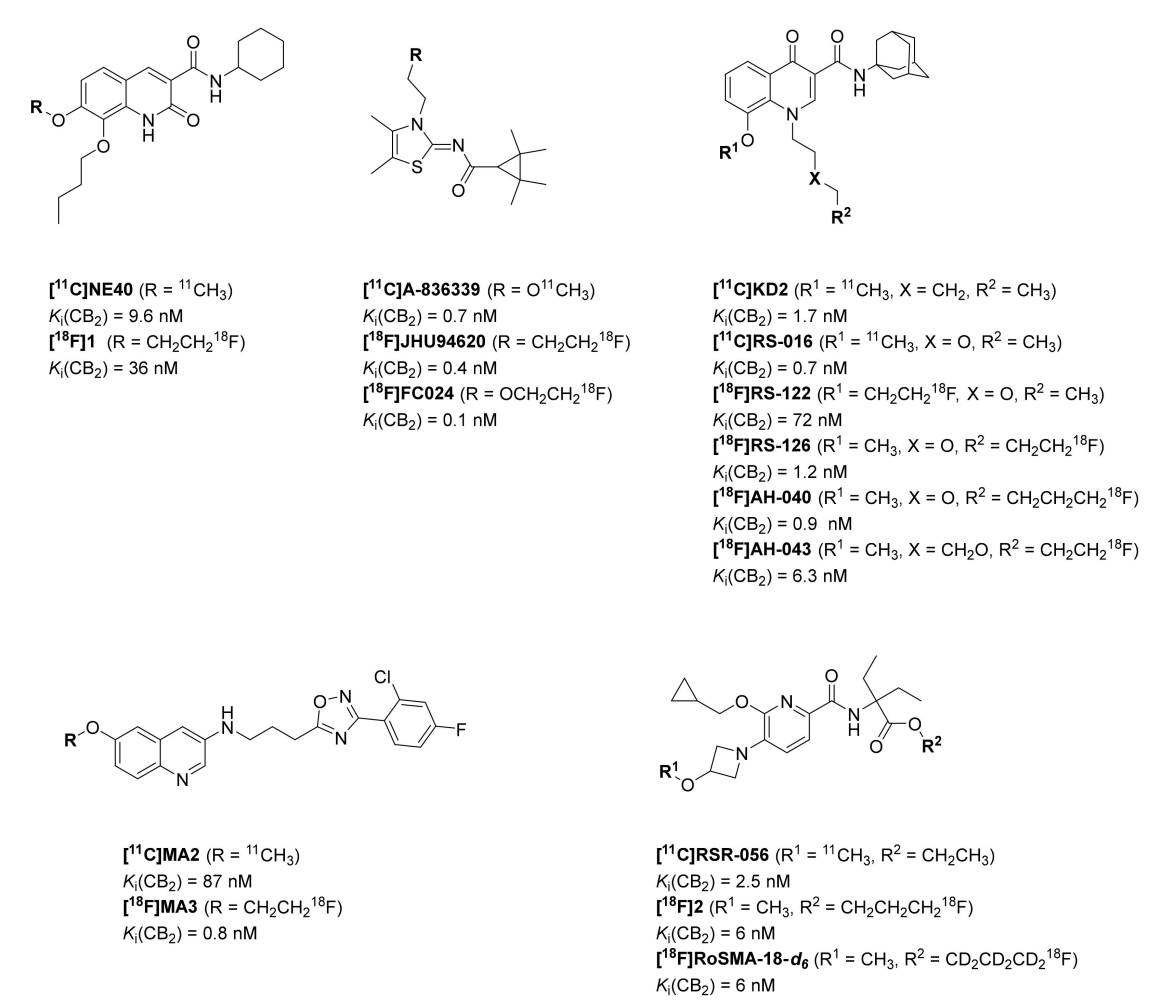
The structure of selected CB2R PET radioligands [42,47].

Despite tremendous efforts, the development of a suitable radiotracer for PET imaging of the CB2R in the brain remains a challenging task [47]. To gain eligibility for consideration as a potential PET radioligand for brain imaging, the first criterion to be fulfilled by a CB2R ligand is high receptor affinity and selectivity, which in many cases is hampered by the homologous similarity between CB1R and CB2R. Another important criterion is a balanced lipophilicity which is usually used as a predicting tool for (1) the ability to cross the blood–brain barrier and (2) non-specific binding. The development of CB2R ligands with optimal lipophilicity as a brain-targeting drug is also challenging due to the highly lipophilic nature of the CB2R binding pocket and its ability to primarily recognise highly lipophilic compounds (e.g., THC has a LogD_7.4_ > 7) [48,49]. Figure 1 shows some of the most representative CB2R radioligands [42,47]. Among them, [^11^C]**NE40** [50,51] is the only CB2R radioligand investigated in humans so far [52]. The suitability of CB2R imaging in pathologic brain conditions was demonstrated by Horti and co-workers by using the 2-aminothiazole [^11^C]**A-836339** [53]. Further structurally related radioligands were developed by Moldovan and co-workers, who designed [^18^F]**JHU94620**, [54,55] and Caille and co-workers, who synthesised [^18^F]**FC0324** [56,57]. However, all these radioligands suffer from low metabolic stability in vivo. A comprehensive work was performed by the group of Ametamey, which reported on a series of ^11^C and ^18^F-labelled quinolinones (Figure 1) [58] and thiophenes (not shown in Figure 1) [59]. Further, labelled 2,3,5-oxodiazoles were prepared by Ahamed [60] and co-workers as derivatives of radioligands earlier reported by Teodoro [61] and co-workers and evaluated for their ability to detect CB2R in vivo. Recently, considerable progress was made by the discovery of the ^11^C- and ^18^F-labelled pyridine radioligands [^11^C]**RSR-056** and [^18^F]**3** [62,63] and, most importantly, the deuterated analogue [^18^F]**RoSMA-18-*d_6_***, [64], which appears to be the most promising CB2R radioligand for PET imaging so far.

**Scheme 1:**
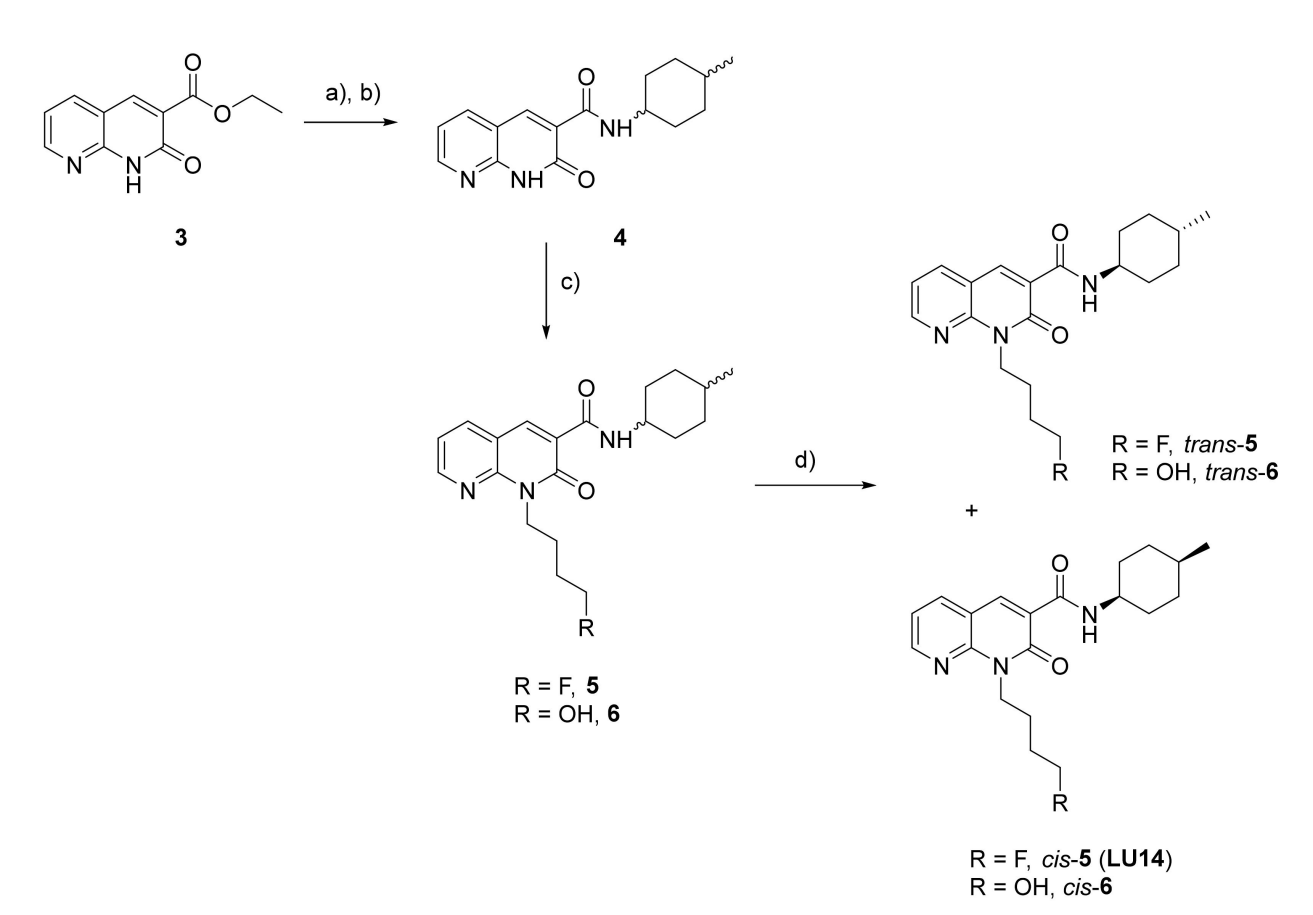
Synthesis of compounds *cis-* and *trans*-**5** and **6**. Reagents and conditions: a) LiOHxH_2_O, THF/MeOH/H_2_O (2:1:1, *v*/*v*/*v*), 60 °C, 2.5 h; b) 4-methylcyclohexylamine, BOP, NEt_3_, DCM, 16 h, rt; c) 1-bromo-4-fluorobutane for *cis*-*/trans*-**5** and 1-bromo-4-butanol for *cis*-*/trans*-**6**, K_2_CO_3_, DMF, 90 °C, 19 h; d) flash chromatography, SiO_2_ eluting with EA/IH 1:9. BOP = [(1*H*-benzo[*d*][1,2,3]triazol-1-yl)oxy]tris(dimethylamino)phosphonium hexafluorophosphate(V) [66].

In this work, we focus on the development and biological evaluation of a radiofluorinated compound belonging to the naphthyridin-2-one class, and we selected compound **5** (Scheme 1) due to the published high affinity and selectivity towards CB2R (*K*_i_ (hCB2R) = 1.3 nM, SI > 700) and its favourable physicochemical properties for targeting the brain [34,65,66]. Compound **5** was characterised as a diastereomeric mixture; however, for similar compounds, a considerably higher binding affinity was described for the *cis* as compared to the *trans* diastereomer [66], and therefore, we pursued the development of the ^18^F-labelled *cis*-**5** (**LU14**, Scheme 1). In order to evaluate in vivo the ability of **LU14** to image CB2R in the brain by PET, we selected a well-established rat model carrying an adeno-associated viral (AAV2/7) vector expressing hCB2R(D80N) at high densities in a striatal region [50,67,68].

## 2. Results and Discussion

### 2.1. Organic Chemistry

The synthesis of the chosen reference compound **5** is depicted in Scheme 1 and was performed based on the synthetic route reported by Lucchesi et al. [66]. In brief, naphtyridin-2-one-3-ethyl ester **3** was condensed with *cis*-/*trans*-4-methylcyclohexylamine under thermal reaction conditions to give amide **4**, which was further *N*-alkylated under alkaline reactions conditions to provide **5**. For compounds similar to **5**, Lucchesi and co-workers [66] reported a strong discrepancy between the binding affinities of the *cis* and *trans* diastereomers towards the CB2R caused by a different binding modality/interaction within the CB2R binding pocket. This encouraged us to perform the diastereomeric separation of **5** achieved by flash chromatography on silica 40–63 Micron eluting with isohexane(45–60)/ethyl acetate (IH/EA), gradient 9:1 to 3:2. The purity determination and identification of the two diastereomers were performed by HPLC, 1D and 2D-NMR spectroscopy (see Supporting Information) and by comparing the chemical shifts with published data.

### 2.2. In Vitro Binding Assay

**Figure 2:**
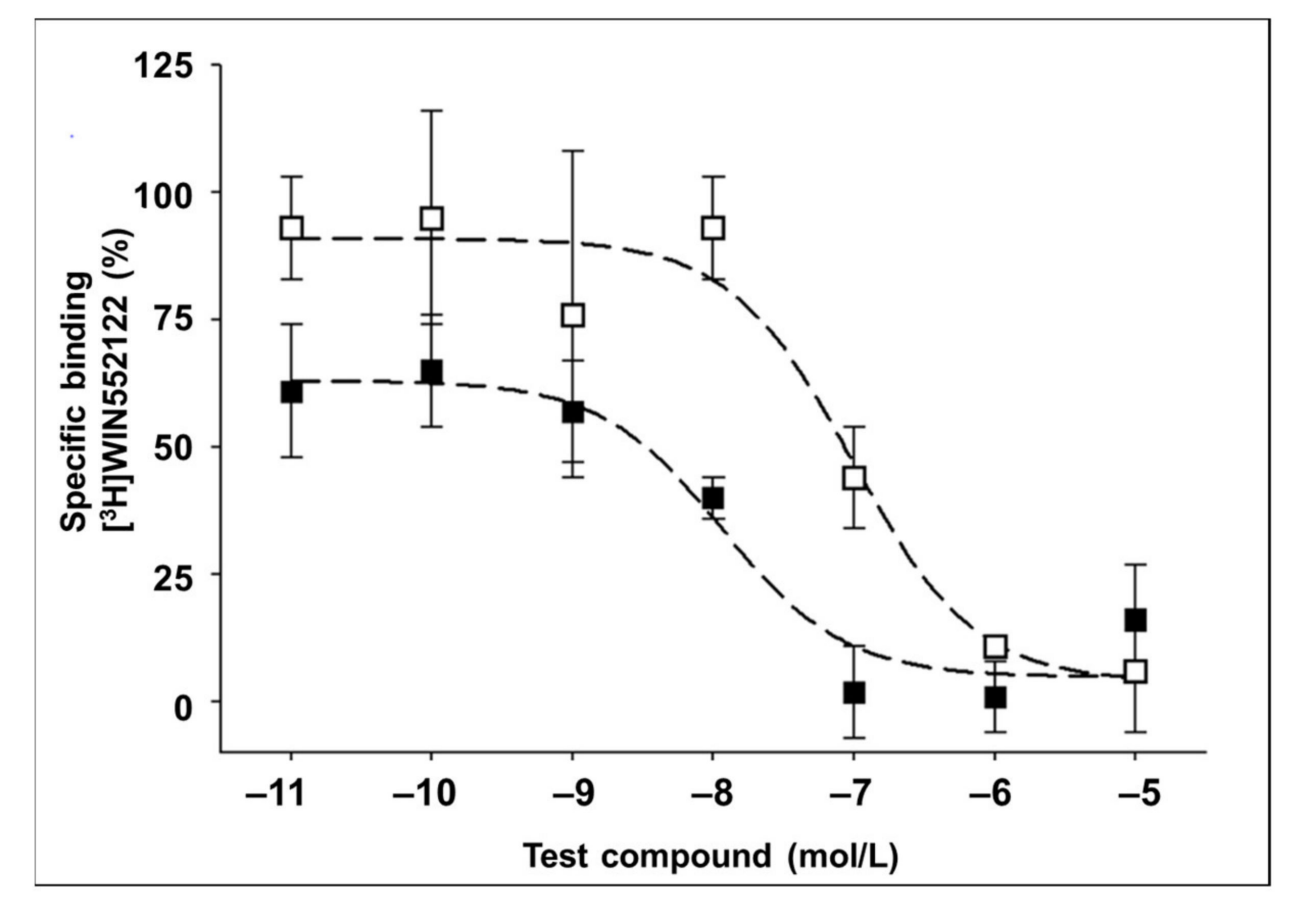
Receptor binding assay to determine the affinity of *cis*-**5** (**LU14**, filled squares) and *trans*-**5** (open squares) for human CB2 receptors. A direct comparison reveals the higher affinity of the *cis*-isomer. Human CB2 receptors were expressed in CHO cells, and affinities were determined by competitive radioligand displacement experiments using [^3^H]WIN552122. Inhibition curves were obtained by non-linear regression of percent specific binding (mean ±SD; one experiment run in triplicate) plotted versus log concentration of the test compound.

In the present work, we evaluated the binding affinity of the separated diastereomers *cis*-**5** and *trans*-**5** towards the CB1R and CB2R and determined the *K*_i_(hCB2R) of 6 nM for *cis*-**5** and of 52 nM for *trans*-**5** (Figure 2).

For **5**, a *K*_i_(hCB2R) of 1.4 nM was reported [66]. The binding affinity of *cis*-*/trans*-**5** was previously determined by Lucchesi and co-workers by using isolated membranes from HEK-293 cells transfected with CB2R and [^3^H]CP-55,940 as a competitor. It is highly likely that the slight difference between the reported and herein determined CB2R binding affinities is related to different experimental protocols. Our data confirm the high affinity of **5** towards the CB2R as well as a nearly 10-fold higher affinity of the *cis* isomer, as reported for similar members of this class of compounds [66]. Consistent with the reported binding affinity for **5**, both diastereoisomers are highly selective towards CB1R (*K*_i_ > 1 µM) [66]. In view of the remarkable affinity and selectivity of *cis*-**5** (**LU14**), the radiofluorination, and subsequently, the pre-clinical characterisation of [^18^F]**LU14** was carried out.

### 2.3. Radiochemistry

The mesylate precursor for radiosynthesis, **7** was prepared with a similar synthetic route as described for the fluorinated compounds, depicted in Scheme 1. Briefly, the carboxamide **4** was coupled with 1-bromo-4-butanol followed by the separation of the diastereomers and the mesylation of the hydroxyl group. For the optimisation of the reaction and the efficient formation of [^18^F]**LU14**, the reaction conditions were varied concerning the amount of precursor **7**, solvent, base and temperature. Choosing a less basic environment by applying TBAHCO_3_^-^ in MeCN at 100 °C resulted in non-isolated radiolabelling yields lower than 10%. Use of K2.2.2./K_2_CO_3_ in DMSO at 150 °C with a very low amount of **7** (1–2 mg) resulted in a two-fold increase of the non-isolated radiolabeling yields.

**Scheme 2:**
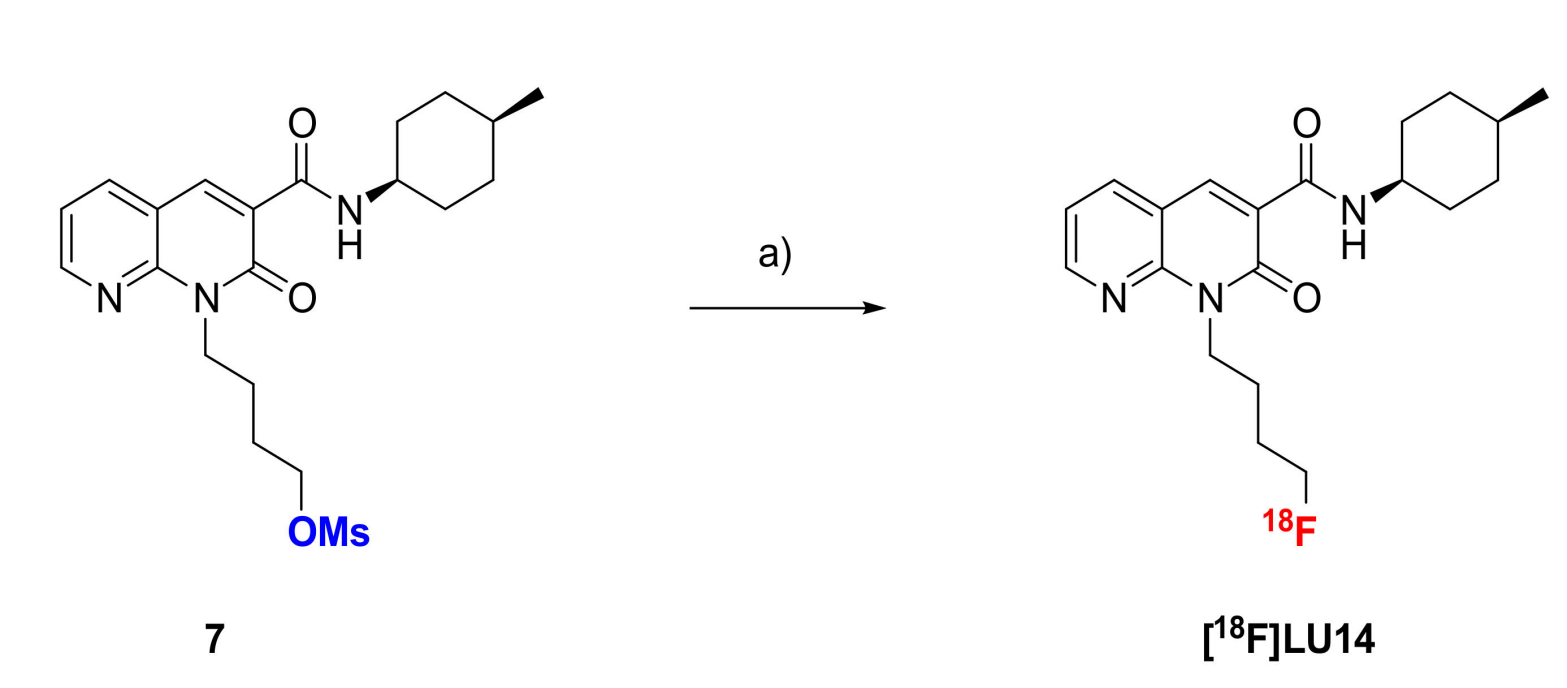
Radiosynthesis of [^18^F]**LU14**. Reagents and conditions: a) [^18^F]K2.2.2., K_2_CO_3_, DMSO, 150 °C 10 min.

**Figure 3:**
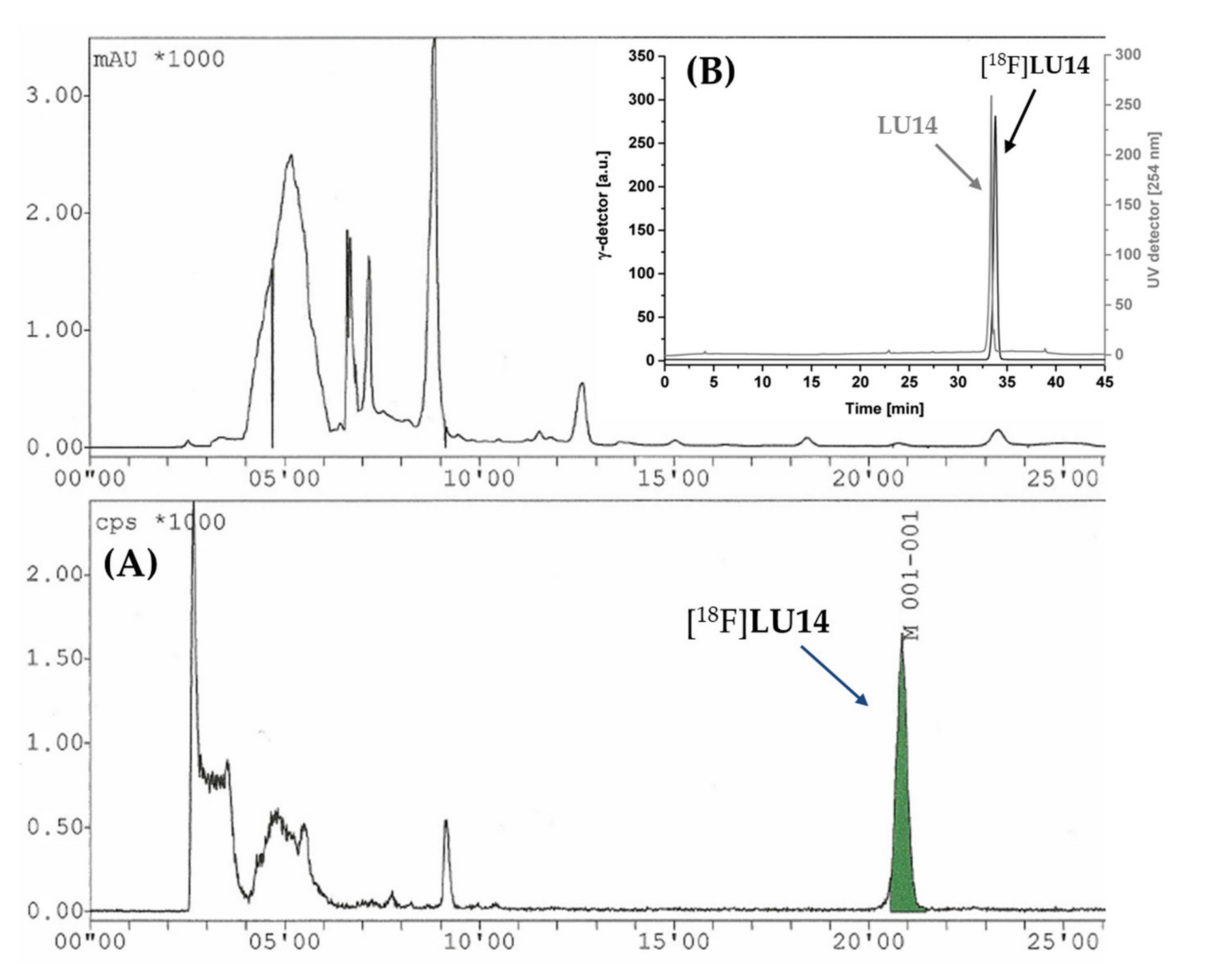
(**A**) Representative semi-preparative radio-HPLC profile of [^18^F]**LU14** (Conditions: Reprosil-Pur 120 C18-AQ (5 µm, 250 × 10 mm), 65% CH_3_CN/20 mM NH_4_OAc aq., 4.2 mL/min) and (**B**) analytical UV- and radio-HPLC chromatogram of [^18^F]**LU14** (solid line, ɣ-trace) spiked with the corresponding reference compound **LU14** (grey line, UV-trace); conditions: Reprosil-Pur C18-AQ (5 µm, 250 × 4.6 mm), 70% CH_3_CN/20 mM NH_4_OAc aq., 1 mL/min. mAU = milli-absorbance unit; cps = counts per second.

Based on these preliminary results, an automated radiosynthesis for [^18^F]**LU14** was developed on a Tracerlab FX_FN_ module as depicted in Scheme 2. The purification of the product was performed by semipreparative HPLC (Figure 3), followed by trapping on a Sep-Pack C18-light cartridge and elution with EtOH. For biological experiments, the solvent was evaporated under a stream of nitrogen at 70 °C until a volume of about 20–30 µL remained. The product was formulated by using 280 µL saline (0.9% aqueous NaCl). The total synthesis time was about 70 min. [^18^F]**LU14** was obtained with 12.6 ± 4.0% radiochemical yield, high radiochemical purity (> 99%) and high molar activity of 200 ± 35 GBq/µmol at the end of the synthesis (*n* = 8). No decomposition of the formulated product was observed within 24 h at room temperature. The identity of the final product [^18^F]**LU14** was confirmed by co-injection with the corresponding reference compound (Figure 3). A logD_7.4_ of 3.15 ± 0.25 was determined by the shake flask method, which is in accordance with the calculated cLogP of 3.0 (ChemDraw Professional 19.0) [69].

### 2.4. In Vitro Evaluation of [^18^F]LU14

**Figure 4:**
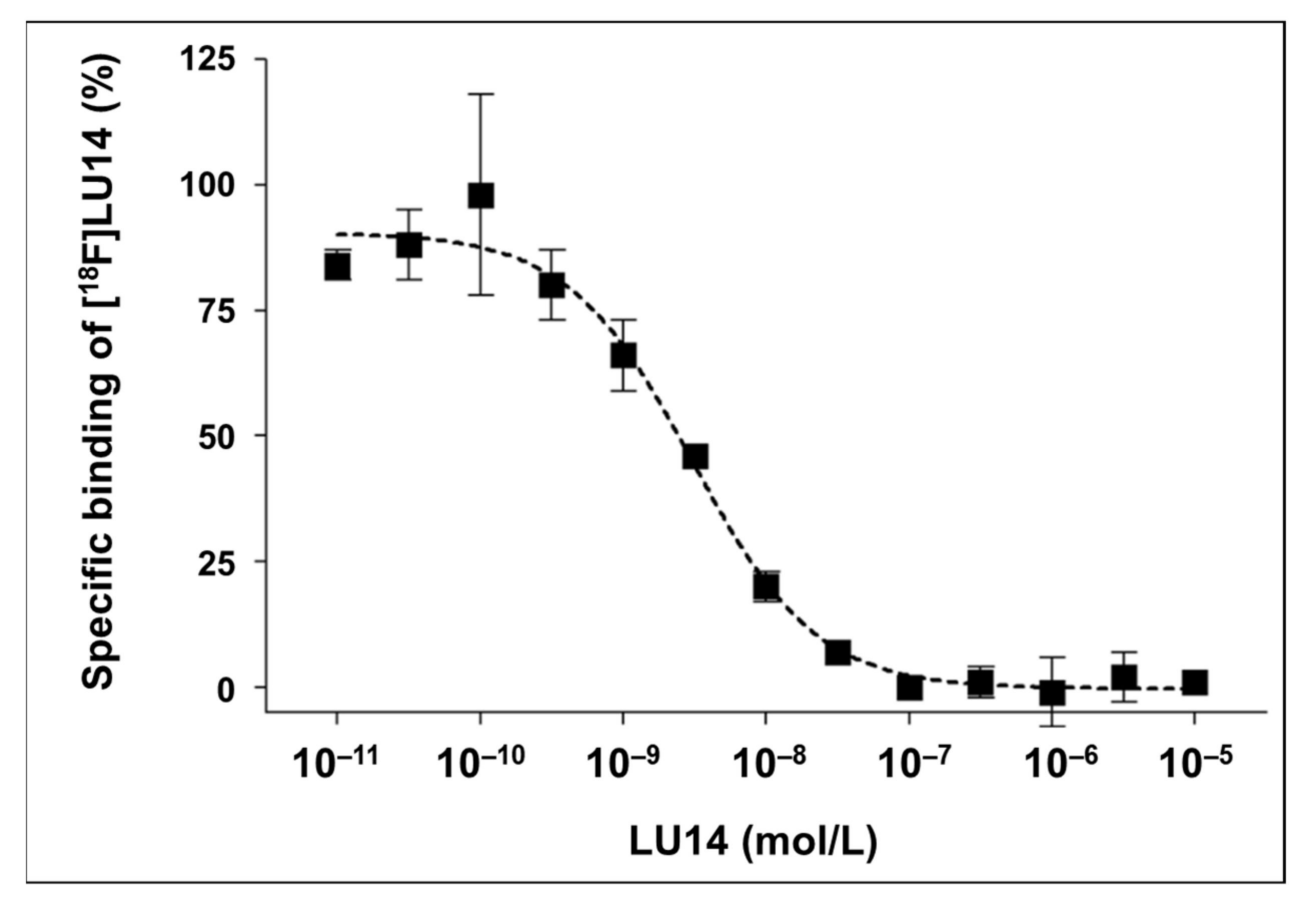
Receptor binding assay to determine the affinity of [^18^F]**LU14** for human CB2 receptors. Human CB2 receptors were expressed in CHO cells, and affinities were determined by competitive radioligand binding experiment using [^18^F]**LU14** at different molar activities. The inhibition curve was obtained by non-linear regression of percent specific binding (mean ±SD; one experiment run in triplicate) plotted versus the log concentration of the test compound.

As a first step to assess the ability of [^18^F]**LU14** to label the CB2R, the equilibrium dissociation constant *K*_D_(hCB2R) was determined by homologues competition using hCB2R-CHO cell membrane homogenates (Figure 4). A value of 2.9 nM was experimentally determined. Next, the in vitro evaluation by autoradiography on spleen tissue was performed (Supplementary Figure S1), as the spleen is regarded as a suitable reference organ, having high densities of CB2R (rat spleen CB2R B_max_ = 0.71 pmol/mg) [70,71]. Cryosections of spleen tissue (10 µm; SPRD rats, female; *n* = 3–4) were incubated with [^18^F]**LU14**. However, as reflected by the images in Supplementary Figure S1, the spleen autoradiography revealed no or low specific binding of [^18^F]**LU14**. The inability to visualise CB2 receptors in rodent spleen tissue was previously reported also for other highly affine CB2R agonists [72,73,74], e.g., [^18^F]**MA3** (Figure 1) [60].

### 2.5. In Vivo Metabolism

The metabolism of [^18^F]**LU14** in vivo was investigated by the use of blood plasma, brain and spleen tissue obtained from female CD-1 mice at 30 min after radiotracer injection.

**Figure 5:**
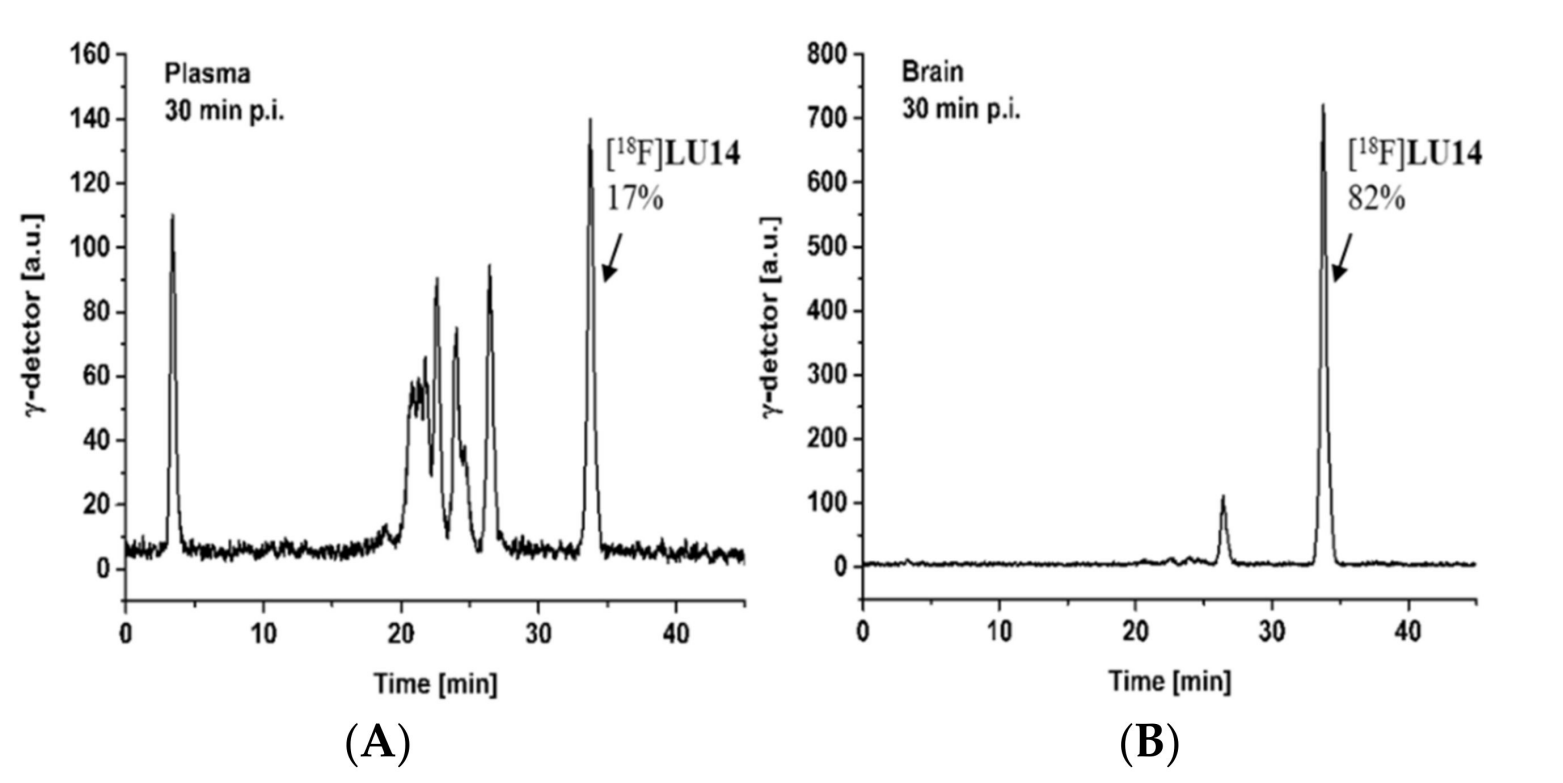
Analytical radio-HPLC chromatograms of the in vivo metabolism of [^18^F]**LU14** at 30 min p.i. (**A**) Plasma samples [extraction with MeOH/H_2_O (9:1); extraction yield: 91%] and (**B**) brain homogenates [extraction with MeOH/H_2_O (9:1); extraction yield: 97%]. HPLC conditions: Reprosil-Pur C18-AQ (250 × 4.6 mm; 5 µm); gradient mode (10-90-10% CH_3_CN/20 mM NH_4_OAc aq.).

As shown in Figure 5, a rather strong metabolic conversion of [^18^F]**LU14** into more polar metabolites was observed. In plasma, only 17 ± 2% (*n* = 3) of unchanged [^18^F]**LU14** was measured. The radioactive signal detected between 2 and 4 min represented about 10% of total activity. It might be caused by fluoride salts formed by the defluorination of the tracer or similar [^18^F]*N*-alkyl chain oxidative degradations [75]. A similar analytical radio-HPLC chromatogram was also observed when investigating the spleen tissue, but with a higher amount of intact [^18^F]**LU14** (50 ± 10% of the total activity detected), which might be a hint of a CB2R-specific retention of the tracer in this organ. In the brain, 82 ± 5% intact [^18^F]**LU14** was detected (Figure 5).

### 2.6. PET Imaging

As the CB2R are not expressed in a healthy brain at a sufficient level to be visualised by PET, the ability of [^18^F]**LU14** to image CB2R expression in the spleen was investigated. Dynamic small animal PET/MR scans with [^18^F]**LU14** under control and blocking conditions were performed in healthy male Wistar rats. The time–activity curves (TACs, Supplementary Figure S2) revealed a rather low uptake in the spleen, comparable to muscle tissue used as reference (SUV ratio spleen-to-muscle of 1.1 to 1.4 between 20 and 60 min p.i.). This is in agreement with the results from the autoradiography study described above. A single blocking experiment (Supplementary Figure S2) revealed comparable AUC_0–60min_ (AUC = area under the curve) values in the spleen for [^18^F]**LU14** in the spleen similar to the control experiments, thus confirming the lack of specific accumulation of [^18^F]**LU14** under these conditions.

**Figure 6:**
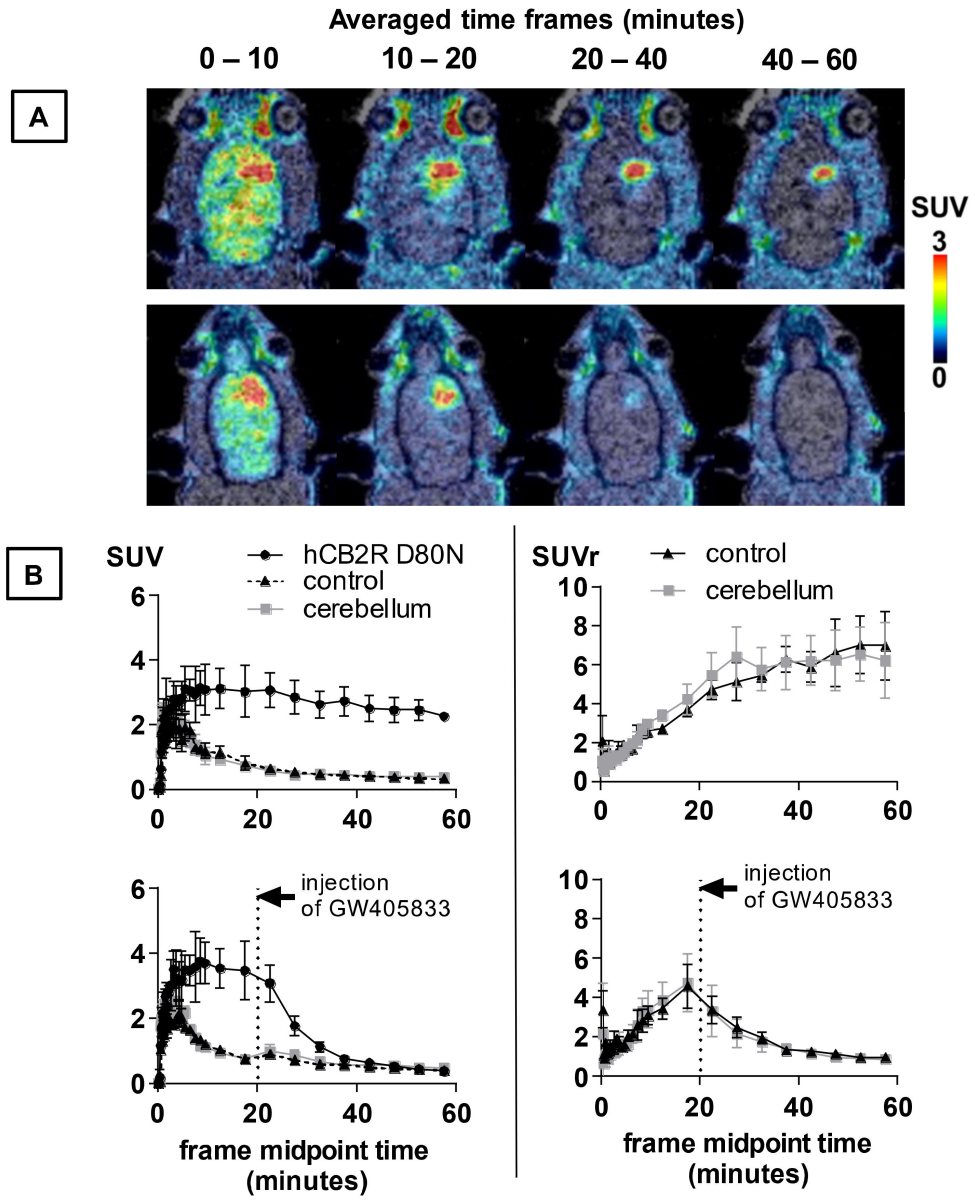
PET imaging of [^18^F]**LU14** in a rat model of local hCB2R overexpression. (**A**) Representative coronal planes showing merged MR and PET images of averaged time frames of the control group (upper row) and displacement study (lower row), (**B**) TACs of the right (hCB2R D80N) and left striatum (contralateral), as well as of the cerebellum on the left side and the SUV hCB2R D80N normalised to contralateral or cerebellum (SUVr), with results of the vehicle treated group in the upper row (*n* = 3) and of the displacement studies where GW405833 (5 mg/kg, *n* = 2) was applied at 20 min. p.i. in the lower row.

In order to evaluate the ability of [^18^F]**LU14** to penetrate the blood–brain barrier and potentially image the human CB2R (hCB2R) in pathological conditions, PET imaging experiments were performed with [^18^F]**LU14** in a rat model with local overexpression of hCB2 receptors in the right striatum, as described by Vandeputte et al. [67] and validated as a suitable model for evaluation of both CB2R agonists and antagonists by Attili [68]. As shown in Figure 6 and Table 1, analysis of the TACs revealed a significantly higher uptake into the striatal region overexpressing the hCB2R(D80N), compared to the contralateral region and the cerebellum after i.v. injection of [^18^F]**LU14**. The initial uptake was followed by a washout with a significantly longer mean residence time (MRT) in the target region compared to non-target regions. Based on these findings, we conclude that (i) these experiments confirm the ability of [^18^F]**LU14** to penetrate the BBB, (ii) that the model clearly reveals a specific uptake of [^18^F]**LU14** under pseudopathological conditions of elevated hCB2R expression and (iii) that, due to the negligible uptake of radioactivity in the cerebellum after i.v. of [^18^F]**LU14** during the time span of the analysis, this region could be used as a reliable reference region in our analysis (Table 2). Taking the aforementioned assumptions into account, the SUVr (hCB2R-to- contralateral ratio) reached a plateau (6.6 ± 1.7 and 7.0 ± 1.7) at 45 min p.i. and the SUVr (hCB2R-to-cerebellum ratio) an earlier plateau (5.8 ± 1.1 to 6.5 ± 1.4) at 30 min p.i. Thus, both reference regions demonstrated favourable kinetics of this tracer to image cerebral CB2R. The uptake of [^18^F]**LU14** in the brain region with CB2R overexpression was about five-fold higher compared to the previously reported radioligand [^18^F]**MA3** [68] and about two-fold higher compared to [^11^C]**NE40** in the same rat model [68]. In order to assess whether the binding of [^18^F]**LU14** to the CB2R is specific and reversible, displacement experiments were performed by the intravenous administration of the known CB2R agonist GW405833 at 20 min p.i (Figure 6B). The amount of activity in the brain region with CB2R overexpression was considerably displaceable by the competitor, as shown by the significant reduction of the AUC_20–60min_ by 73% (compared to the contralateral region) and 76% (compared to the cerebellum) (Table 1). In summary, these experiments demonstrate that [^18^F]**LU14** is a specific CB2R radioligand with reversible receptor binding, favourable kinetics and high potential to be used for the detection of cerebral CB2R overexpression in pathological conditions like neuroinflammation or brain cancer.

## 3. Materials and Methods

### 3.1. Chemistry

#### 3.1.1. General Information

All chemicals and reagents were purchased from commercially available sources and used without further purification. Moisture-sensitive reactions were conducted under dry argon with oven-dried glassware and anhydrous solvents. Reaction progress was monitored by thin-layer chromatography (TLC) using Alugram SIL G/UV_254_ pre-coated plates (Macherey-Nagel; Düren, Germany). The spots were identified by using a UV lamp or by dipping the plates into a potassium permanganate solution (3 g KMnO_4_, 20 g K_2_CO_3_, 0.25 mL glacial acid, 300 mL water). For the purification of products, flash column chromatography was used with silica gel 40–63 μm (VWR International Chemicals, Darmstadt, Germany). The purity of all the tested compounds was ≥95% as determined by an LC-MS system including a DAD detector (Dionex Ultimate 3000, Thermo Fisher Scientific Inc., Waltham, MA, USA) system incorporating a LPG-3400SD pump, a WPS-3000 TSL autosampler, a TCC-3000SD column compartment, a DAD 3000 diode array detector and an MSQ 3000 low-resolution mass spectrometer (Thermo Fisher Scientific Inc.; Waltham, MA, USA), column: Reprosil-Pur Basic HD (150 × 3 mm; 3 µm; Dr. Maisch GmbH; Ammerbuch, Germany), gradient: 10-90-10% MeCN/20 mM NH_4_OAc_aq._ (*v/v*), run time: 15 min, flow rate: 0.6 mL/min, UV-detection: 254 nm]. ^1^H-, ^13^C- and ^19^F-NMR spectra were recorded on VARIAN Mercury plus (300 MHz for ^1^H-NMR, 75 MHz for ^13^C-NMR, 282 MHz for ^19^F-NMR, Agilent Technologies, Palo Alto, CA, USA) and BRUKER DRX-400 (400 MHz for ^1^H-NMR, 100 MHz for ^13^C-NMR, 377 MHz for ^19^F-NMR, Bruker, Billerica, MA, USA); chemical shifts (δ) in parts per million (ppm) are related to internal tetramethylsilane, and coupling constants (*J*) are given with 0.1 Hz. High-resolution mass spectra (HRFT-MS) were recorded on an FT-ICR APEX II spectrometer (Bruker Daltonics; Bruker Corporation; Billerica, MA, USA) using electrospray ionisation (ESI).

#### 3.1.2. Chemical Synthesis

Ethyl-2-oxo-1,2-dihydro-1,8-naphthyridine-3-carboxylate (**3**) [66]. Diethylmalonate (9.40 mL, 9.87 g, 61.6 mmol, 2.5 equiv) and Piperidine (6 mL, 5.16 g, 60.6 mmol, 2.5 equiv) were added to a solution of 2-aminopyridine-3-carbaldehyde (3.01 g, 24.6 mmol, 1 equiv) in 60 mL EtOH. The reaction mixture was stirred for 19 h under reflux. The yellow suspension was filtered at RT, washed with EtOH and concentrated under reduced pressure to give the light-yellow solid **3** in 81% yield (4.34 g, 19.9 mmol). ^1^H NMR (400 MHz, DMSO-d_6_) δ = 1.30 (t, J = 7.1 Hz, 3 H), 4.28 (q, J = 7.1 Hz, 2 H), 7.29 (dd, J = 7.8, 4.7 Hz, 1 H), 8.27 (dd, J = 7.9, 1.8 Hz, 1 H), 8.49 (s, 1 H), 8.60 (dd, J = 4.7, 1.8 Hz, 1 H), 12.43 (s, 1 H).

2-Oxo-1,2-dihydro-1,8-naphthyridine-3-carboxylic acid [66]. Ethyl-2-oxo-1,2-dihydro-1,8-naphthyridine-3-carboxylate (**3**, 1.01 g, 4.64 mmol, 1 equiv) and LiOH·H_2_O (0.575 g, 13.7 mmol, 3 equiv) were dissolved in 20 mL of a mixture of THF:MeOH:H_2_O (2:1:1, *v*/*v*/*v*) and stirred for 2.5 h at 60 °C. Half of the solvent was removed under reduced pressure. Water and hydrochloric acid (3M) were added until a pH of 3 was reached. After filtration and the washing of the precipitation with hydrochloric acid (1M), the light-yellow solid was dried to yield 96% (0.843 g, 4.43 mmol) of the acid, which was used in the next step without purification.

*N*-(4-Methyl)-2-oxo-1,2-dihydro-1,8-naphthyridine-3-carboxamide (**4**) [66]. To a solution of 2-oxo-1,2-dihydro-1,8-naphthyridine-3-carboxylic acid (0.052 g, 0.27 mmol, 1 equiv) and BOP (0.156 g, 0.35 mmol, 1.3 equiv) in 10 mL DCM, triethylamine (0.11 mL, 0.08 g, 0.79 mmol, 2.9 equiv) and *cis*-/*trans*-4-methylamine (0.035 mL, 0.03 g, 0.26 mmol, 1 equiv) were added, and the reaction mixture was stirred for 40.5 h at RT. The reaction mixture was purified by column chromatography (silica, DCM→DCM:MeOH (9.5:0.5) and DCM→DCM:MeOH (9.5:0.5, *v/v*)). The solvent was removed under reduced pressure and, after drying, the light-green solid was obtained in 41% (0.032 g, 0.112 mmol) yield. ^1^H NMR (400 MHz, CDCl_3_): *δ* = 0.92 (d, ^3^*J* = 6.5 Hz, 3 H), 0.99 (d, ^3^*J* = 6.6 Hz, 3 H), 1.11 (qd, ^3^*J* = 13.2, 3.4 Hz, 2 H), 1.33 (m, 5H), 1.55 (ddq, ^3^*J* = 9.8, 6.5, 3.3 Hz, 1 H), 1.66 (ddt, ^3^*J* = 16.2, 9.2, 4.2 Hz, 4 H), 1.77 (d, ^3^*J* = 13.3 Hz, 2 H), 1.85 (dd, ^3^*J* = 10.7, 6.1 Hz, 2 H), 2.08 (dd, ^3^*J* = 13.2, 3.7 Hz, 2 H), 3.92 (tdt, ^3^*J* = 11.8, 8.0, 4.1 Hz, 1 H), 4.30 (dq, ^3^*J* = 7.9, 3.9 Hz, 1 H), 7.39 (dd, ^3^*J* = 7.8, 4.7 Hz, 2 H), 8.19 (m, 2 H), 8.83 (s, 2 H), 8.95 (d, ^3^*J* = 3.1 Hz, 2 H), 9.43 (d, ^3^*J* = 8.0 Hz, 1 H), 9.85 (d, ^3^*J* = 7.9 Hz, 1 H); ^13^C NMR (101 MHz, CDCl_3_): *δ* = 21.9, 22.4, 29.9 (s, 2 C), 30.2 (s, 2 C), 31.4, 32.1, 33.0 (s, 2 C), 34.0 (s, 2 C), 45.6, 49.1, 119.7 (s, 2 C), 124.5, 124.7, 139.3, 139.4, 143.1, 143.1, 149.4 (s, 2 C), 150.9 (s, 2 C), 161.4, 161.5, 162.8, 163.0; HRMS (ESI+) *m/z* for C_16_H_19_N_3_NaO_2_^+^ [M + Na]^+^ 308.1380, calcd 308.1375.

1-(4-Fluorbutyl)-N-(4-methyl)-2-oxo-1,2-dihydro-1,8-naphthyridine-3-carboxamide (**5**) [66]. Compound **4** (0.162 g, 0.59 mmol, 1 equiv) was placed under inert atmosphere in 50 mL DCM. Cs_2_CO_3_ (0.518 g, 1.6 mmol, 2.8 equiv) was added, and the reaction mixture was stirred for 45 min at RT. 1-Brom-4-fluorobutane (0.240 mL, 0.350 g, 2.3 mmol, 4 equiv) was added, and the reaction mixture was stirred for 16 h at 50 °C. The crude product was extracted with DCM and water, and, subsequently, the organic layer was dried over MgSO_4_ and filtered. The obtained residue was purified by column chromatography (silica, EtOAc and isohexane (1:9→7:3)). The solvents were removed under reduced pressure, and 32% of the white solid (*trans*-**5**), as well as 30% of the white solid (*cis-***5**), was obtained. *trans*-**5**: ^1^H NMR (400 MHz, CDCl_3_): *δ* = 0.91 (d, ^3^*J* = 6.5 Hz, 3 H), 1.11 (m, 2 H), 1.33 (m, 2 H), 1.40 (m, 1 H), 1.76 (m, 2 H), 1.88 (m, 4 H), 2.08 (m, 2 H), 3.92 (tdt, ^3^*J* = 11.8, 8.0, 4.1 Hz, 1 H), 4.53 (dt, ^3^*J* = 47.6, 6.4, 5.9 Hz, 2 H)), 4.63 (t, ^3^*J* = 7.3 Hz, 2 H). 7.28 (dd, ^3^*J* = 7.8, 4.7 Hz, 1 H), 8.09 (dd, ^3^*J* = 7.8, 1.8 Hz, 1 H), 8.71 (dd, ^3^*J* = 4.7, 1.9 Hz, 1 H), 8.88 (s, 1 H), 9.63 (s, 1 H); ^13^C NMR (101 MHz, CDCl_3_): *δ* = 22.4, 24.1 (d, ^3^*J* = 5.3 Hz), 28.1 (d, ^2^*J* = 20.1 Hz), 32.1, 33.1 (s, 2 C), 34.1 (s, 2 C), 41.6, 49.0, 83.9 (d, ^1^*J* = 165.0 Hz), 115.1 (s, 0.25-), 119.2, 123.3, 138.7, 142.1, 149.7, 152.1, 162.0, 162.7; ^19^F NMR (377 MHz, CDCl_3_): *δ* = −218.2; HRMS (ESI+) *m/z* for C_20_H_26_FN_3_NaO_2_^+^ [M + Na]^+^ 382.1925, calcd 382.1901; *cis-***5**: ^1^H NMR (400 MHz, CDCl_3_): *δ* = 0.98 (d, ^3^*J* = 6.6 Hz, 3 H), 1.31 (m, 3 H), 1.55 (ddp, ^3^*J* = 9.8, 6.4, 3.0 Hz, 1 H), 1.66 (m, 4 H), 1.87 (m, 6 H), 4.26 (dp, ^3^*J* = 8.6, 4.5 Hz, 1 H), 4.53 (dt, ^3^*J* = 47.2, 5.7 Hz, 2 H), 4.65 (dd, ^3^*J* = 8.5, 6.2, Hz 2 H). 7.28 (dd, ^3^*J* = 7.8, 4.8 Hz, 1 H), 8.08 (dd, ^3^*J* = 7.8, 1.9 Hz, 1 H), 8.71 (dd, ^3^*J* = 4.7, 1.8 Hz, 1 H), 8.88 (s, 1 H), 10.00 (d, ^3^*J* = 7.7 Hz, 1 H); ^13^C NMR (101 MHz, CDCl_3_): *δ* = 21.6, 24.0 (d, ^3^*J* = 5.4 Hz), 28.1 (d, ^2^*J* = 20.0 Hz), 29.7 (s, 2 C), 30.3 (s, 2 C), 31.2, 41.5, 45.9, 83.9 (d, ^1^*J* = 165 Hz), 115.1, 119.1, 123.4, 138.7, 141.9, 149.7, 152.0, 162.0, 162.7; ^19^F NMR (377 MHz, CDCl_3_): *δ* = −13.4; HRMS (ESI+) *m/z* for C_20_H_26_FN_3_NaO_2_^+^ [M + Na]^+^ 382.1923, calcd 382.1901.

1-(4-Hydroxybutyl)-N-(4-methylcyclohexyl)-2-oxo-1,2-dihydro-1,8-naphthyridine-3-carboxamid (**6**) [66]. Compound **4** (0.162 g, 0.59 mmol, 1 equiv) was placed under inert atmosphere in 50 mL DCM, and Cs_2_CO_3_ (0.518 g, 1.6 mmol, 2.8 equiv) was added, and the reaction mixture was stirred for 45 min at RT. 1-Brom-4-butanol (0.240 mL, 0.350 g, 2.3 mmol, 4 equiv) was added, and the reaction mixture was stirred for 16 h at 50 °C. The crude product was extracted with DCM and water, and, subsequently, the organic layer was dried over MgSO_4_ and filtered. The obtained residue was purified by column chromatography (silica, EtOAc: isohexane (2:8)→EtOAc) and dried to give *trans*-**6** in 20% yield (0.041 g, 0.12 mmol) and *cis*-**6** in 26% yield (0.053 g, 0.148 mmol) as white solids. *trans*-**6:** ^1^H NMR (400 MHz, CDCl_3_): *δ* = 0.92 (d, ^3^*J* = 6.5 Hz, 3 H), 1.11 (m, 2 H), 1.33 (m, 3 H), 1.75 (m, 2 H), 1.99 (m, 6 H), 3.49(t, ^3^*J* = 6.6 Hz, 2 H), 3.91 (tdt, ^3^*J* = 11.9, 8.1, 4.1 Hz, 1 H), 4.61 (t, ^3^*J* = 7.2 Hz, 2 H), 7.28 (dd, ^3^*J* = 7.8, 4.7 Hz, 1 H), 8.07 (dd, ^3^*J* = 7.8, 1.9 Hz, 1 H), 8.70 (dd, ^3^*J* = 4.7, 1.9 Hz, 1 H), 8.87 (s, 1 H), 9.60 (d, ^3^*J* = 8.0 Hz, 1 H); ^13^C NMR (101 MHz, CDCl_3_): *δ* = 22.4, 26.9, 30.4, 32.2, 33.1, 33.3, 34.1 (s, 2 C), 41.1, 49.0, 115.1, 119.2, 123.3, 138.6, 142.1, 149.8, 152.2, 162.0, 162.7; HRMS (ESI+) *m/z* for C_20_H_28_N_3_O_3_^+^ [M + H]^+^ 340.2029, calcd 340.2025.; *cis*-**6**: ^1^H NMR (400 MHz, CDCl_3_): *δ* = 0.98 (d, ^3^*J* = 6.6 Hz, 3 H), 1.30 (m, 3 H), 1.55 (ddp, ^3^*J* = 9.8, 6.5, 3.0 Hz, 1 H), 1.67 (m, 3 H), 1.84 (m, 2 H), 1.98 (m, 4 H), 3.50(t, ^3^*J* = 6.6 Hz, 2 H), 4.6 (tq, ^3^*J* = 8.4, 4.3 Hz, 1 H), 4.63 (t, ^3^*J* = 7.6, 7.1 Hz, 2 H), 7.27 (dd, ^3^*J* = 7.8, 4.7 Hz, 1 H), 8.07 (dd, ^3^*J* = 7.8, 1.9 Hz, 1 H), 8.70 (dd, ^3^*J* = 4.7, 1.9 Hz, 1 H), 8.86 (s, 1 H), 9.97 (d, ^3^*J* = 7.8 Hz, 1 H); ^13^C NMR (101 MHz, CDCl_3_): *δ* = 21.7, 26.9, 29.7, 30.3, 30.4, 31.2, 33.3 (s, 2 C), 41.0, 45.8, 115.1, 119.2, 123.4, 138.5, 141.9, 149.8, 152.1, 162.0, 162.7; HRMS (ESI+) *m/z* for C_20_H_28_N_3_O_3_^+^ [M + H]^+^ 340.2036, calcd 340.2025.

### 3.2. Radiochemistry

#### 3.2.1. Automated Radiosynthesis of [^18^F]LU14

Remote-controlled radiosynthesis was performed using a Synchrom R&D EVO III automated synthesiser (Elysia-Raytest, Straubenhardt, Germany). Briefly, [^18^F]fluoride (4–6 GBq) was trapped on a Waters QMA cartridge and eluted with a solution containing 5.6 mg K222 and 40 μL K_2_CO_3_ dissolved in a mixture of H_2_O/MeCN (1 mL, 1:4, *v/v*) into the reaction vessel and dried via azeotropic distillation. An additional 1.5 mL of dried MeCN was added. After complete dryness, a solution containing 1 mg of precursor **7** in 800 µL DMSO was added, and the reaction mixture was stirred at 150 °C for 10 min. Upon cooling to 40 °C, the reaction mixture was diluted with 4 mL H_2_O, and the resulting solution was transferred to the semi-preparative HPLC. [^18^F]**LU14** was collected in the HPLC collection vial containing 40 mL of H_2_O and trapped in the Sep-Pak^®^ C18 light cartridge (Waters GmbH, Eschborn, Germany). The cartridge was washed with 2 mL H_2_O, and [^18^F]**LU14** was eluted with 1.2 mL EtOH. This ethanolic solution was transferred outside of the shielded cell, and the solvent was evaporated at 70 °C in a gentle stream of nitrogen for 5–10 min, and [^18^F]**LU14** was reconstituted in isotonic saline solution for further biological characterisation. Total synthesis time was about 70 min.

#### 3.2.2. Quality Control

Radio-thin-layer chromatography was performed on Alugram SIL G/UV_254_ pre-coated plates (Macherey-Nagel, Düren, Germany) with IH:EA (1:1, *v*/*v*). The plates were exposed to storage phosphor screens (BAS IP MS 2025 E, GE Healthcare Europe GmbH, Freiburg, Germany) and recorded using the Amersham Typhoon RGB Biomolecular Imager (GE Healthcare Life Sciences, Solingen, Germany). Images were quantified with the ImageQuant TL8.1 software (GE Healthcare Life Sciences, Solingen, Germany).

Analytical chromatographic separations were performed on a JASCO LC-2000 system, incorporating a PU-2080*Plus* pump, AS-2055*Plus* auto-injector (100 μL sample loop), and a UV-2070*Plus* detector coupled with a γ-detector (GABI Star, Raytest Isotopenmessgeräte GmbH, Straubenhardt, Germany). Data analysis was performed with the Galaxie chromatography software (Agilent Technologies, Santa Clara, CA, USA) using the chromatograms obtained at 254 nm.

Radiochemical yield, radiochemical purity and analyses of plasma and brain samples were assessed via reversed-phase HPLC (RP-HPLC) in gradient mode (0–10 min: 10% MeCN/20 mM NH_4_OAc_aq._, 10–30 min: 10%→90% MeCN/20 mM NH_4_OAc_aq._, 30–35 min: 90% MeCN/20 mM NH_4_OAc_aq._, 35–36 min: 90%→10% MeCN/20 mM NH_4_OAc_aq._ 36–45 min: 10% MeCN/20 mM NH_4_OAc_aq._).

The molar activity was determined using analytical radio-HPLC with a Reprosil-Pur C18-AQ column (250 × 4.6 mm, 5 μm) and 62% MeCN/20 mM NH_4_OAc_aq._ as eluent at a flow rate of 1 mL·min^−1^ and UV detection at 224 nm.

#### 3.2.3. Determination of Lipophilicity (LogD_7.4_)

Log*D*_7.4_ of [^18^F]**LU14** was experimentally determined in *n*-octanol/phosphate-buffered saline (PBS; 0.01 M, pH 7.4) at room temperature by the shake-flask method. The measurement was performed twice in triplicate [69].

### 3.3. Biological Experiments

All studies involving animals were carried out according to the national laws on the protection of animals and were approved by the responsible authorities (Landesdirektion Sachsen, No. DD24.1–5131/446/19; TVV 18/18).

#### 3.3.1. Determination of Binding Affinities by Homogenate Assays

The binding affinities towards CB_1_ and CB_2_ receptors were determined according to a protocol published previously [54]. In brief, membrane preparations obtained from CHO cell lines stably transfected with either human CB_1_ (hCB_1_-CHO; obtained from Euroscreen, Gosselies, Belgium) or human CB_2_ (hCB_2_-CHO; obtained from Paul L. Prather, Department of Pharmacology and Toxicology, College of Medicine, University of Arkansas for Medical Sciences, Fayetteville, AR, USA) were used along with [^3^H]SR141716A (1554 GBq/mmol; PerkinElmer Life and Analytical Sciences, Rodgau, Germany) and [^3^H]WIN55212-2 (6438 GBq/mmol; PerkinElmer Life and Analytical Sciences, Rodgau, Germany), resp., as target-specific radioligands. The incubations were performed in 50 mM TRIS-HCl, pH 7.4, supplemented with 0.1% bovine serum albumin (BSA), 5 mM MgCl_2_, and 1 mM EDTA for 90 min at room temperature. Various concentrations of test compounds were administered, and the non-specific binding was determined in the presence of 10 μM **56**. The values of bound activity were used to determine affinity (*K*_i_) values using non-linear regression analysis (GraphPad Prism 2.01, v9, San Diego, CA, USA) and based on the *K*_D_ values of [^3^H]**56** determined by saturation experiments [54].

#### 3.3.2. In Vitro Autoradiography

The in vitro autoradiographic experiments were performed according to a previously published protocol [76]. In brief, 10 µm cryosections of rat spleen (female SPRD rat, 10–12 weeks) were incubated in binding buffer (50 mM TRIS-HCl, pH 7.4, 5% bovine serum albumin (BSA), 5 mM MgCl_2_, 1 mM EDTA) with [^18^F]**LU14** alone (total binding) or with co-administered GW405833 (CB_2_-selective partial agonist) for 1 h at room temperature. Afterwards, the samples were washed, exposed to imaging plates (Fuji Photo Film, Co. Ltd., Tokyo, Japan), and eventually scanned using a HD-CR 35 scanner (raytest Isotopenmessgeraete GmbH, Straubenhardt, Germany). The scan data were visualised and processed by computer-assisted microdensitometry (Aida version 2.31, raytest Isotopenmessgeräte GmbH, Straubenhardt, Germany).

#### 3.3.3. Quantification of Radiometabolites

About 30 MBq [^18^F]**LU14** dissolved in about 150 µL isotonic saline was administered intravenously as a bolus in the tail vein of an awake female CD-1 mice weighing about 33 g (*n* = 3). At 30 min p.i., the animals were anaesthetised and blood was withdrawn by retrobulbar bleeding using glass capillaries. Immediately afterwards, the animals were euthanised by cervical dislocation, and released urine was sampled. Blood plasma was obtained from the whole blood sample by centrifugation (2 min, 8000 rpm, room temperature). In addition, the brain and spleen were isolated and homogenised in 1 mL demineralised water on ice (1000 rpm, 10 strokes; glass vessel, PTFE plunger; Potter S, B. Braun Biotech International, Göttingen, Germany).

The samples were further processed for subsequent radio-chromatographic analyses. Two consecutive extractions were performed as duplicates for plasma and brain determinations. Plasma, brain and spleen samples were added to an ice-cold MeOH/H_2_O mixture (9:1, *v/v*). The samples were vortexed for 3 min, incubated on ice for 5 min and centrifuged at 10,000 rpm for 5 min. Supernatants were collected, and the precipitates were re-dissolved in 100 µL of extraction solvent, and the extraction procedure was repeated. The activities of supernatants and precipitates were measured in a γ-counter (1480 WIZARD, Fa. Perkin Elmer, Turku, Finland), and the extraction efficiencies were calculated as the ratio of radioactivity in the supernatant to the radioactivity in the original sample (supernatant + precipitate). The supernatants from both extractions were combined, concentrated at 70 °C under argon stream up to a remaining volume of 100 µL, and subsequently analysed by analytical radio-HPLC with a gradient system (see quality control section).

#### 3.3.4. PET Studies

In vivo biodistribution of [^18^F]**LU14** in rats was assessed by dynamic small animal PET (Nanoscan, Mediso, Budapest, Hungary) 60 min recordings, followed by T1-weighted (GRE, TR/TE = 15.0/2.4 ms, 252/252, FA = 25°) MR imaging with whole-body coils for anatomical correlation and attenuation correction. For the uptake studies into the spleen three male Wistar Hun rats (Janvier Laboratories, Le Genest Saint Isle, France) weighing 268 ± 56 g were used. For the uptake studies into the brain, five female Wistar rats weighing 263 ± 13 g carrying the stereotactically injected AAV 2/7-CaMKII0.4-intron-hCB2R D80N (right striatum) and AAV2/7-CaMKII0.4-intron-3flag-eGFP (contralateral, left striatum). Animals were initially anaesthetised with 5% isoflurane and placed on a thermostatically heated animal bed where anaesthesia was maintained with 2% isoflurane in 60% oxygen/38% room air. They were pre-treated by i.v. injections of vehicle solution only, containing DMSO: Kolliphor EL: saline in a composition of 1:2:7 (control group) or GW405833 (1.5 mg/kg bodyweight for spleen-scan experiments and 5 mg/kg for the displacement experiments with local overexpression of hCB2R) 10 min prior [^18^F]**LU14** administration. [^18^F]**LU14** (22.4 ± 3.5 MBq) was injected i.v. into the lateral tail vein (bolus within 5 s) at the start of the PET acquisition. List-mode PET data were binned as a series of attenuation-corrected sinogram frames (12 × 10 s, 6 × 30 s, 5 × 60 s and 10 × 300 s) and were reconstructed by ordered subset expectation maximisation (OSEM3D) with four iterations, six subsets and a voxel size of 0.4 mm^3^ (Nucline v2.01, Mediso, Hungary). The analysis of reconstructed datasets was performed with PMOD software (v4.103, PMOD Technologies LLC, Zurich, Switzerland). Non-parametrical analyses of achieved time-activity curves (TACs) were performed with Microsoft Excel to determine the time to peak, the TAC peak value and the area-under-the-zero-momentum curve (AUC):

(1) $$AUC_{0-t\left(x\right)}=\int_{0}^{t\left(x\right)}c\left(radioactivity\right)\;\times \;dt$$

where c (radioactivity) is expressed as a standardised uptake value normalised to the bodyweight in g (SUV), the area-under-first-momentum curve (AUMC):

(2) $$AUMC_{0-t\left(x\right)}=\int_{0}^{t\left(x\right)}t\;\times \;c\left(radioactivity\right)\;\times \;dt$$

and the mean residence time (MRT):

(3) $$MRT=\frac{AUMC_{0-t\left(x\right)}}{AUC_{0-t\left(x\right)}}$$

GraphPad Prism (v9, San Diego, CA, USA) was used for graphical presentation.

## 4. Conclusions

To evaluate the suitability of the naphtyridin-2-one-3-carboxamide class of compounds to target CB2R in the brain, we have chosen the literature-known compound *cis*-*/trans*-**5** and developed [^18^F]**LU14** in stereochemically pure form. The first biological evaluation of [^18^F]**LU14** revealed low nanomolar binding affinity towards human CB2R and adequate metabolic stability in mouse with only trace amounts of radiometabolites detected in the brain at 30 min after radioligand injection. PET experiments in a rat model of human CB2R overexpression revealed that [^18^F]**LU14** is able to reach and reversibly label cerebral CB2R, demonstrating its potential as a radiotracer for detecting CB2R alterations in pathologic conditions.

## Figures and Tables

**Table 1:** Non-compartmental analysis of TACs in the region of local overexpression of the hCB2R(D80N) in the right striatum of rats, the contralateral region and the cerebellum after i.v. injection of [^18^F]**LU14** (*n* = 3).

TAC Parameter	hCB2R(D80N)	Contralateral	*p*-Value	Cerebellum	*p*-Value
time to peak (min)	7.4 ± 2.8	3.8 ± 1.6	0.063	1.6 ± 1.4	0.016
TAC peak value (SUV)	3.3 ± 0.6	2.2 ± 0.4	0.024	2.7 ± 0.0	0.067
AUC (SUV × min)	155 ± 28	42.6 ± 6.8	0.001	41.1 ± 5.6	0.001
AUMC (SUV × min^2^)	4349 ± 701	854 ± 165	0.001	819 ± 132	0.001
MRT (min)	28.0 ± 0.4	20.0 ± 1.1	<0.001	19.9 ± 1.2	<0.001

Mean ±SD; *p*-value—one-sided Student’s *t*-test.

**Table 2:** Uptake of [^18^F]**LU14** into the hCB2R overexpressing right striatum normalised to the contralateral contralateral site or cerebellum with (*n* = 2) or without (*n* = 3) displacement by i.v. injected GW405833 (5 mg/kg bodyweight) at 20 min after radiotracer administration expressed as area under the curve before (AUC_0–20min_) and after (AUC_20–60min_) injection of the interventional drug.

hCB2R D80N-to-	Treatment (5 mg/kg GW405833, i.v.)	AUC_0–20min_ (CI_95%_) in SUVr × min	AUC_40–60min_ (CI_95%_) in SUVr × min
contralateral	vehicle (10 min pior tracer)	40 (37 to 42)	210 (190 to 231)
	displacement (20 min after tracer)	48 (41 to 54)	56 (50 to 62) −73.4%
cerebellum	vehicle (10 min prior tracer)	44 (39 to 48)	215 (190 to 241)
	displacement (20 min after tracer)	49 (40 to 59)	51 (42 to 60) −76.3%

Mean; 95% confidence interval (CI_95%_).

## Data Availability

The data that support the findings of this study are available from the corresponding author upon reasonable request.

## Supplementary Materials

The following are available online at https://www.mdpi.com/article/10.3390/ijms22158051/s1.
