# Supplementary material for: Development of [18F]LU14 for PET Imaging of Cannabinoid Receptor Type 2 in the Brain
Source: Int J Mol Sci. 2021 Jul 28;22(15):8051. doi: 10.3390/ijms22158051 (PMC8347709; doi:10.3390/ijms22158051)
Supplement: Supplementary file 1 [file ijms-22-08051-s001.zip › ijms-1293361-supplementary.pdf]

*Supplementary Material*

# Development of [$^{18}$F]LU14 for PET Imaging of Cannabinoid Receptor Type 2 in the Brain

**Rodrigo Teodoro [1], Daniel Gündel [1], Winnie Deuther-Conrad [1], Lea Ueberham [1], Magali Toussaint [1], Guy Bormans [2], Peter Brust [1,3] and Rareş-Petru Moldovan [1,*]**

[1] Helmholtz-Zentrum Dresden-Rossendorf (HZDR), Institute of Radiopharmaceutical Cancer Research, Department of Neuroradiopharmaceuticals, Research site Leipzig, 04318 Leipzig, Germany; r.teodoro@hzdr.de (R.T.); d.guendel@hzdr.de (D.G.); w.deuther-conrad@hzdr.de (W.D.-C.); uelea@web.de (L.U.); m.toussaint@hzdr.de (M.T.); p.brust@hzdr.de (P.B.)

[2] Radiopharmaceutical Research, Department of Pharmaceutical and Pharmacological Sciences, KU Leuven, BE-3000 Leuven, Belgium; guy.bormans@kuleuven.be

[3] The Lübeck Institute of Experimental Dermatology, University Medical Center Schleswig-Holstein, 23562 Lübeck, Germany

* Correspondence: r.moldovan@hzdr.de, Tel.: +49-3412341794634

**Content:**

1. NMR characterization of compound *trans*-**5** and *cis*-**5** (**LU14**)
2. Rat spleen autoradiography
3. Spleen TAC of [$^{18}$F]**LU14**

*Trans*-**5** $^1$H NMR:

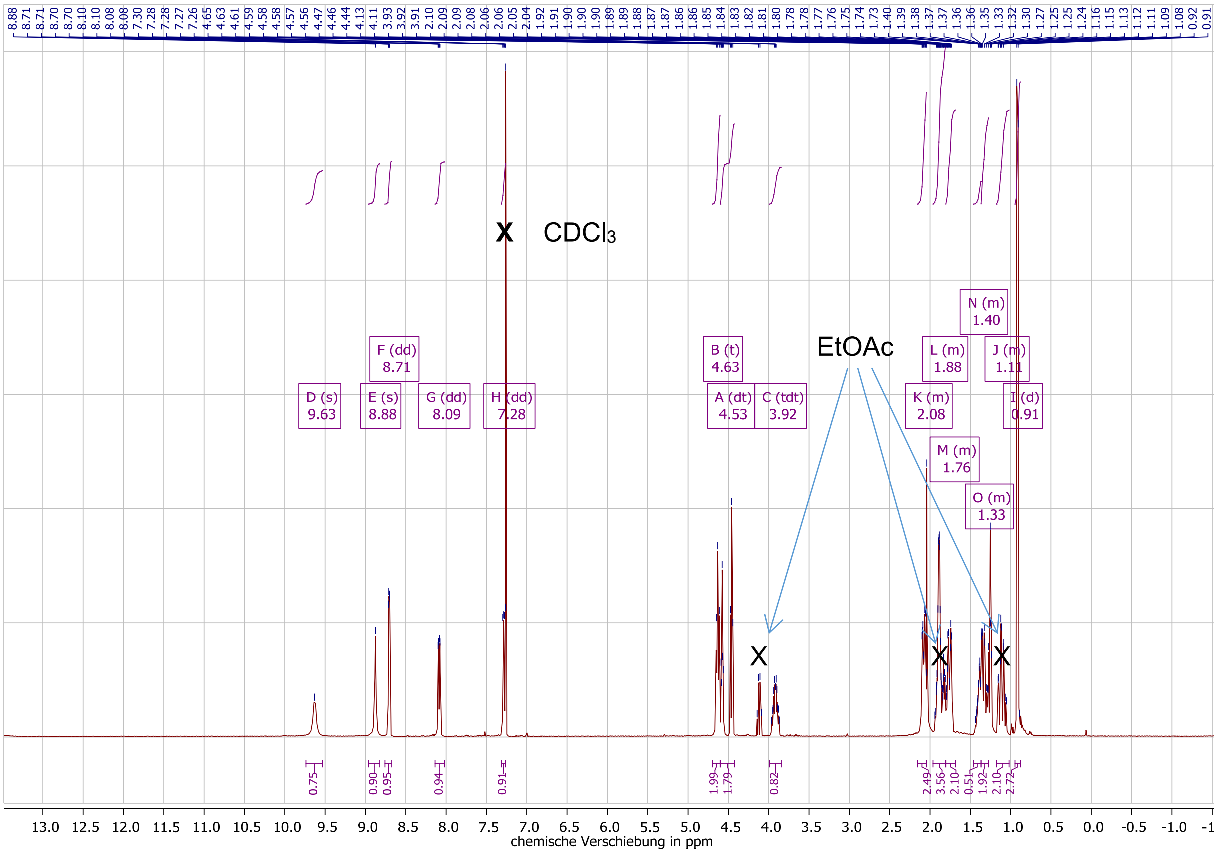

*Trans*-**5** $^{19}$F-NMR:

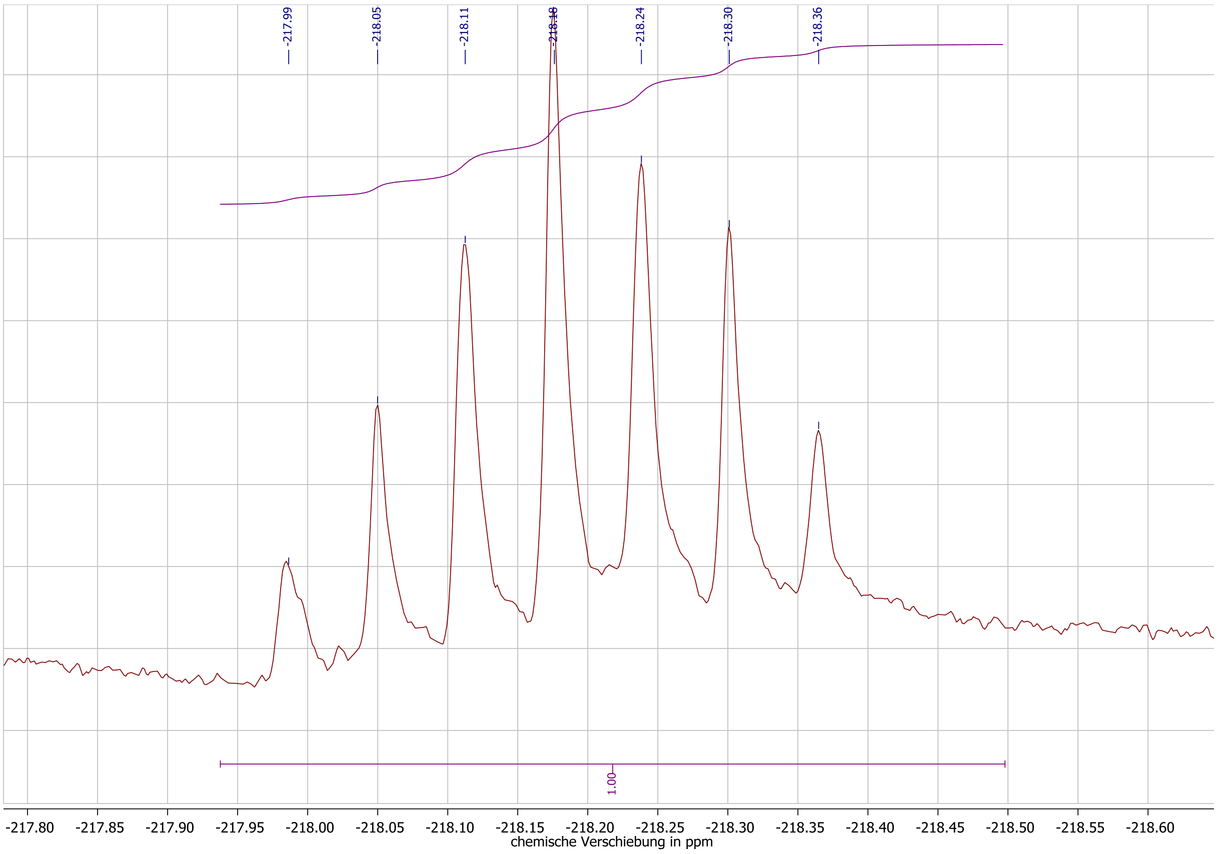


*Trans*-**5** $^{13}$C-NMR:

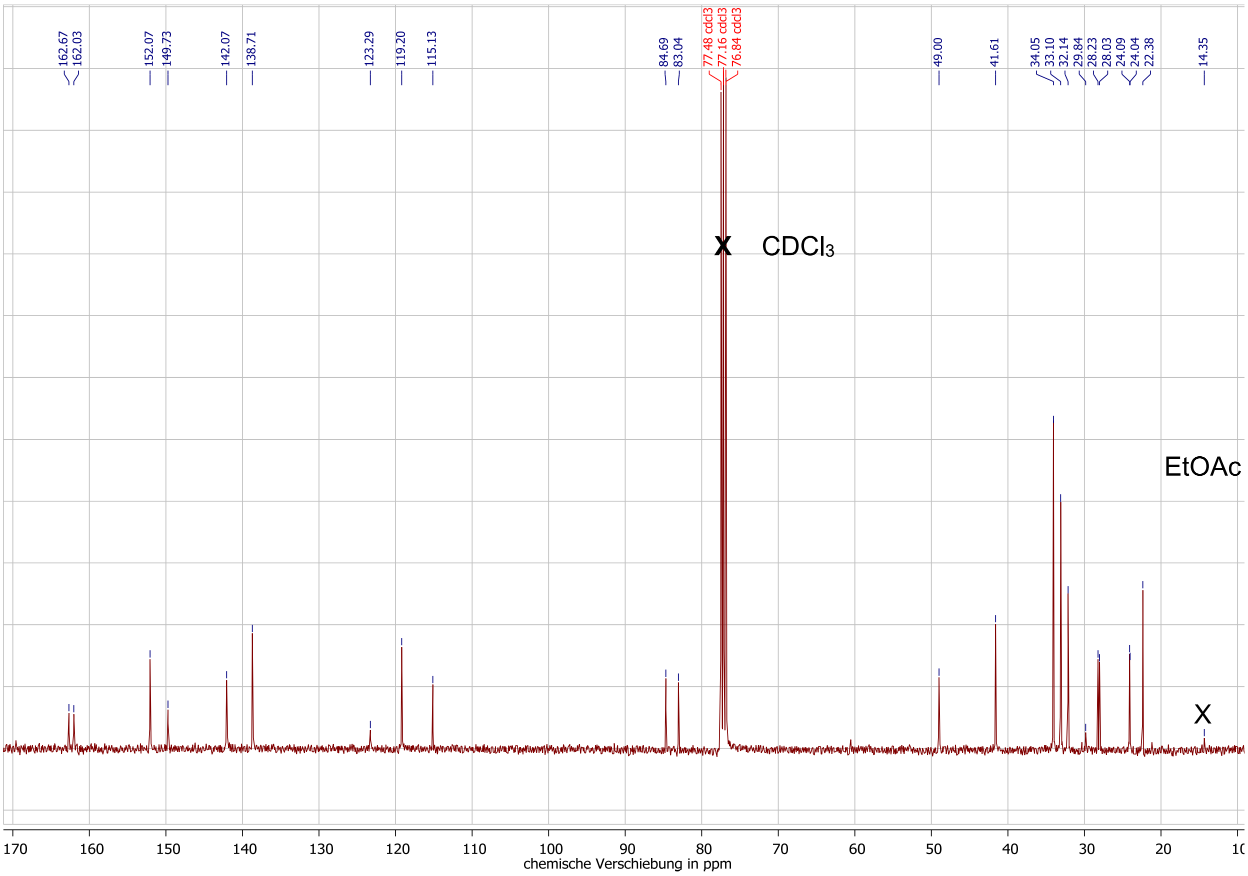

*Trans*-**5** $^{13}$C-APT:

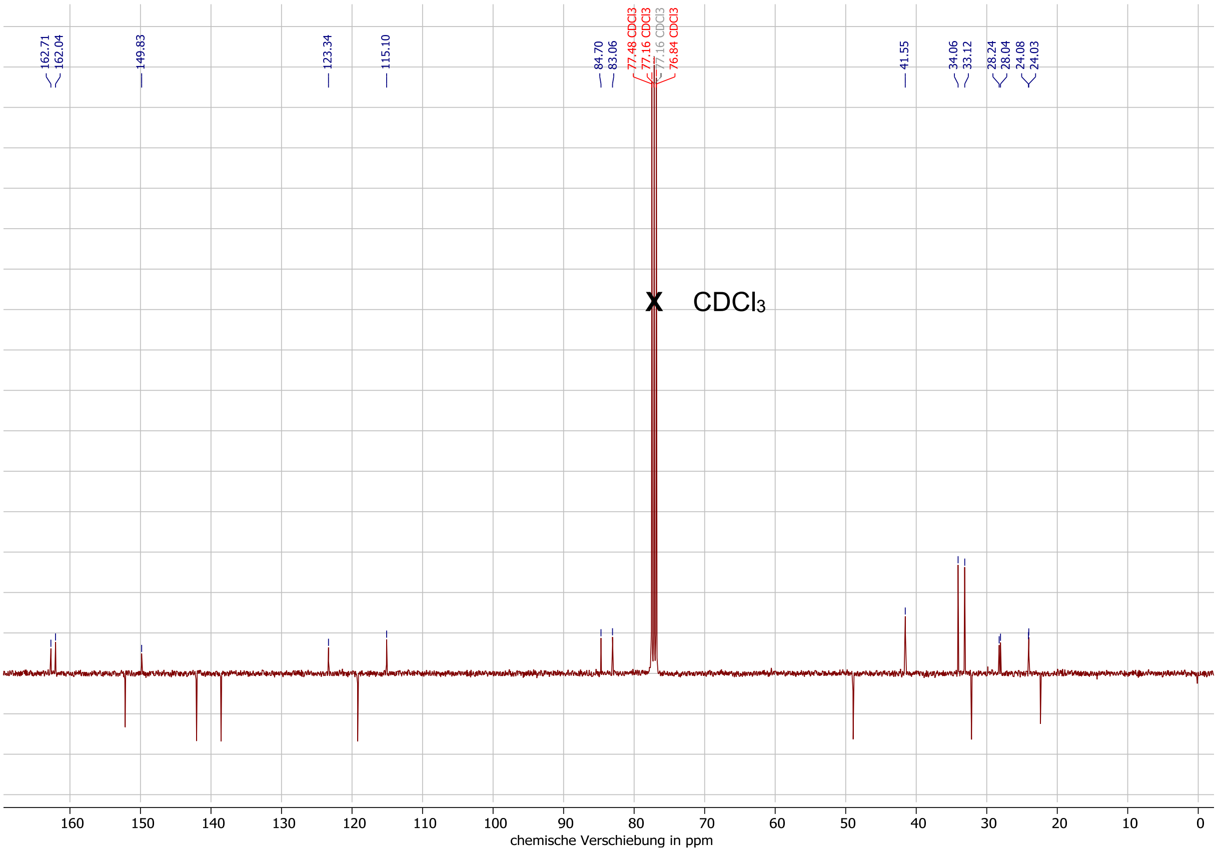


*Trans*-**5** 2D-NMR: COSY:

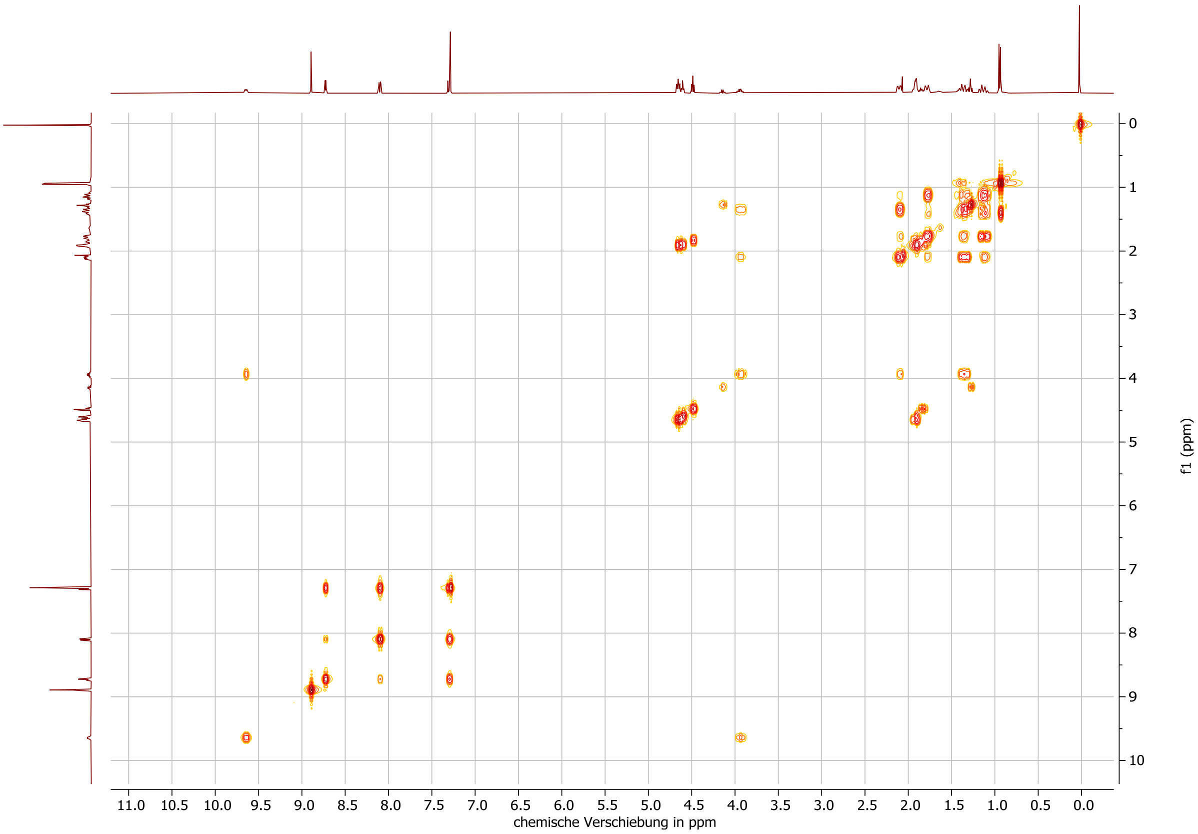

*Trans*-**5** 2D-NMR: NOESY:

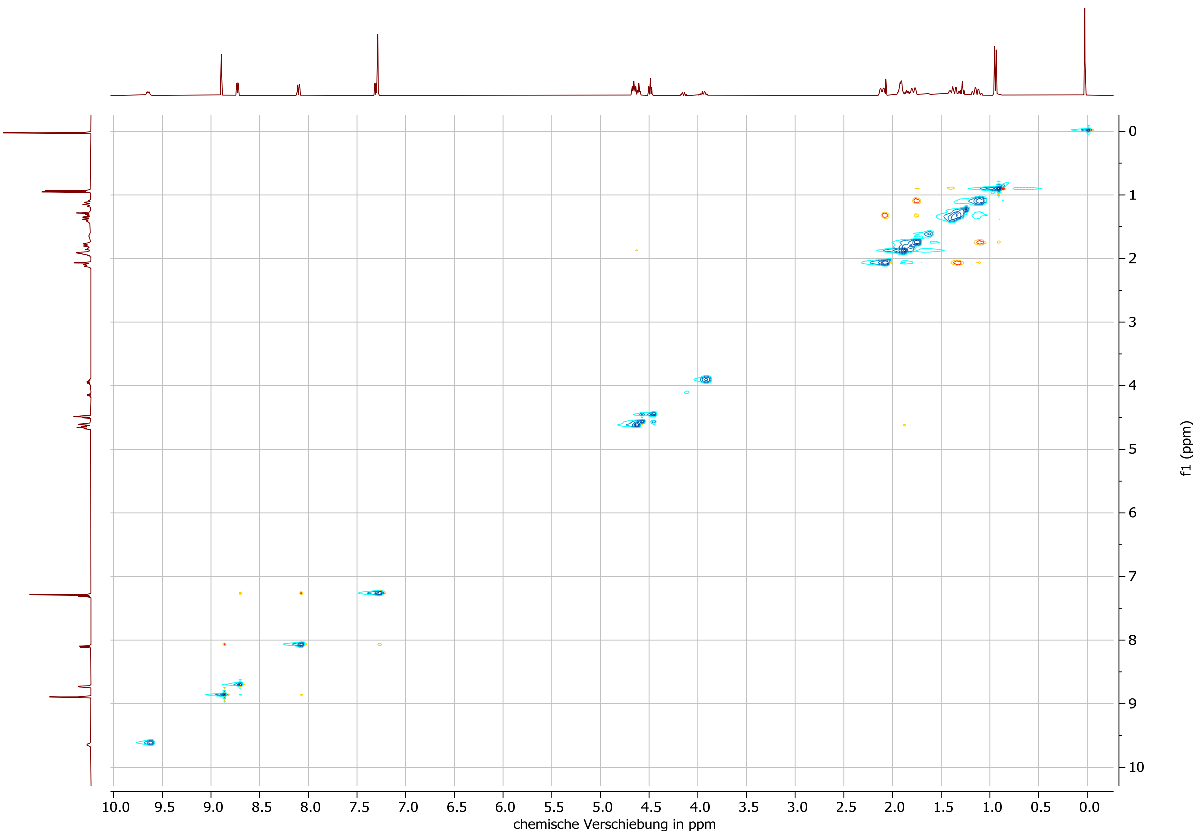


*Trans*-**5** 2D-NMR: HMBC:

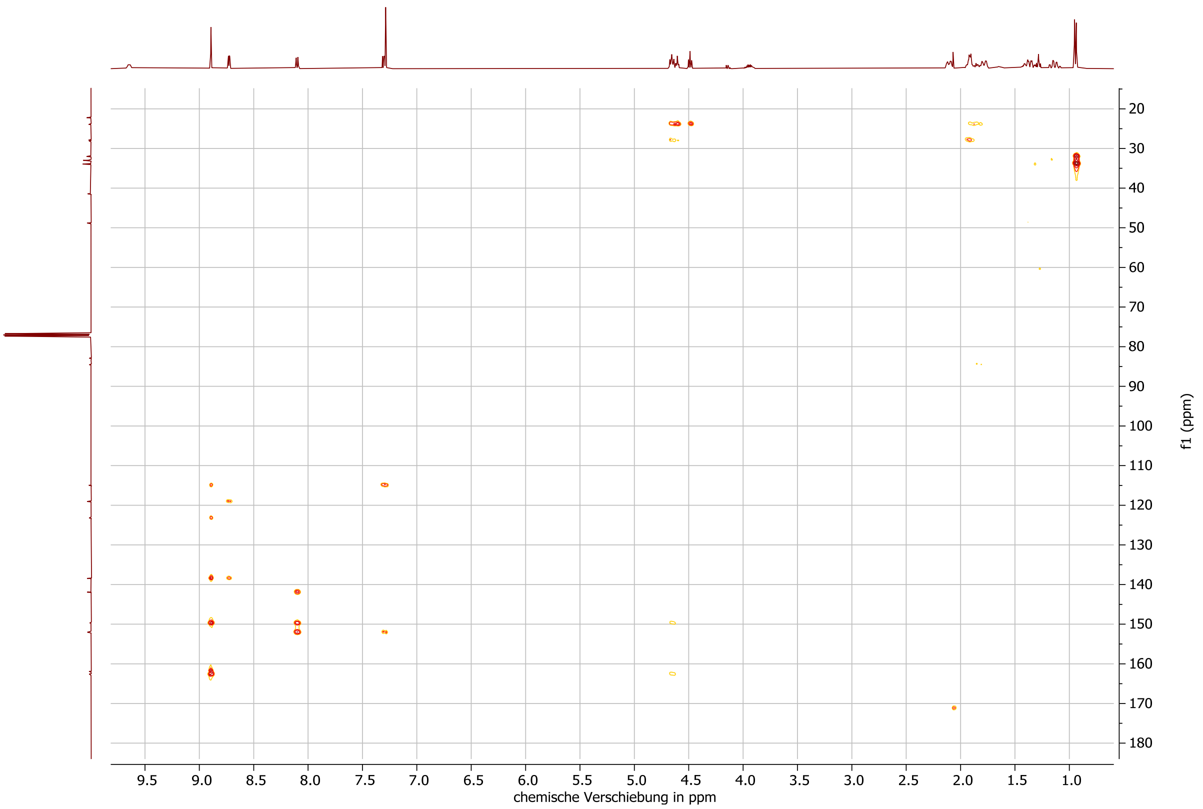

*Trans*-**5** 2D-NMR: HSQC:

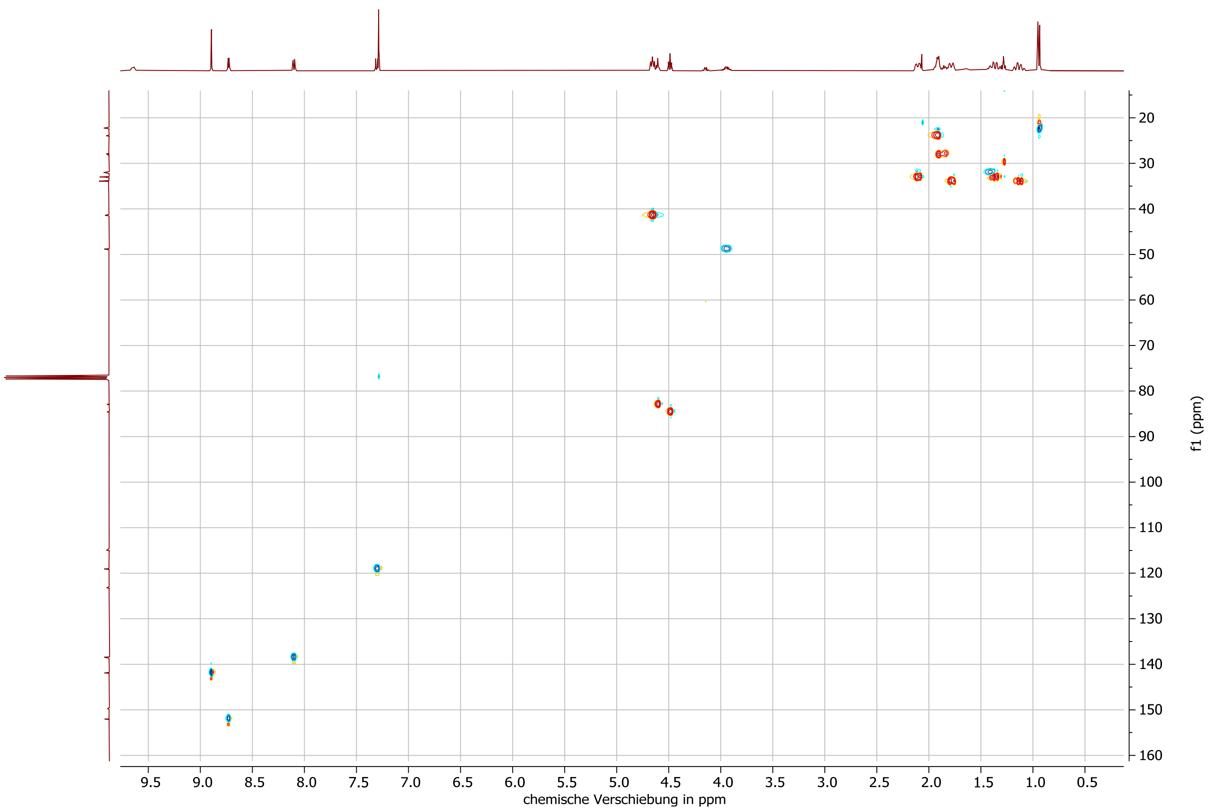


**LU14** $^{1}$H-NMR:

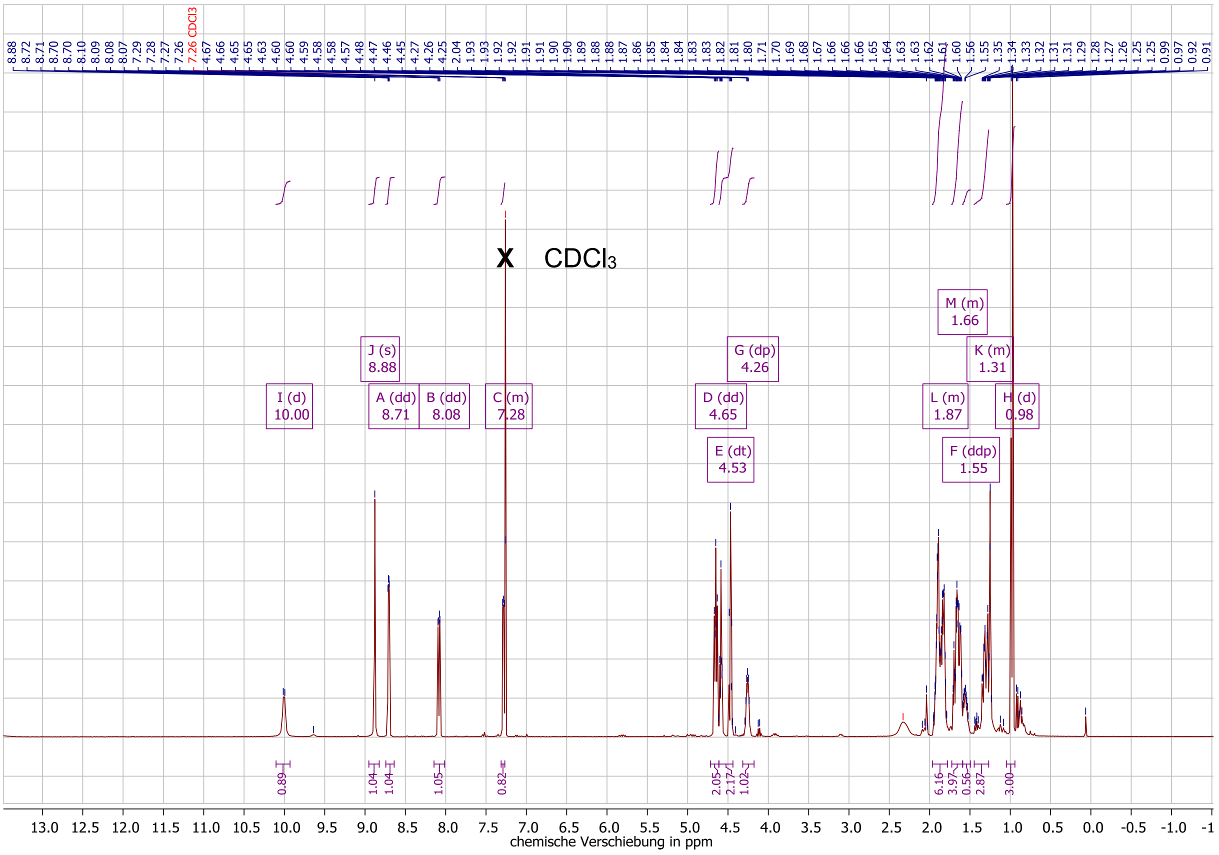

**LU14** $^{13}$C-NMR:

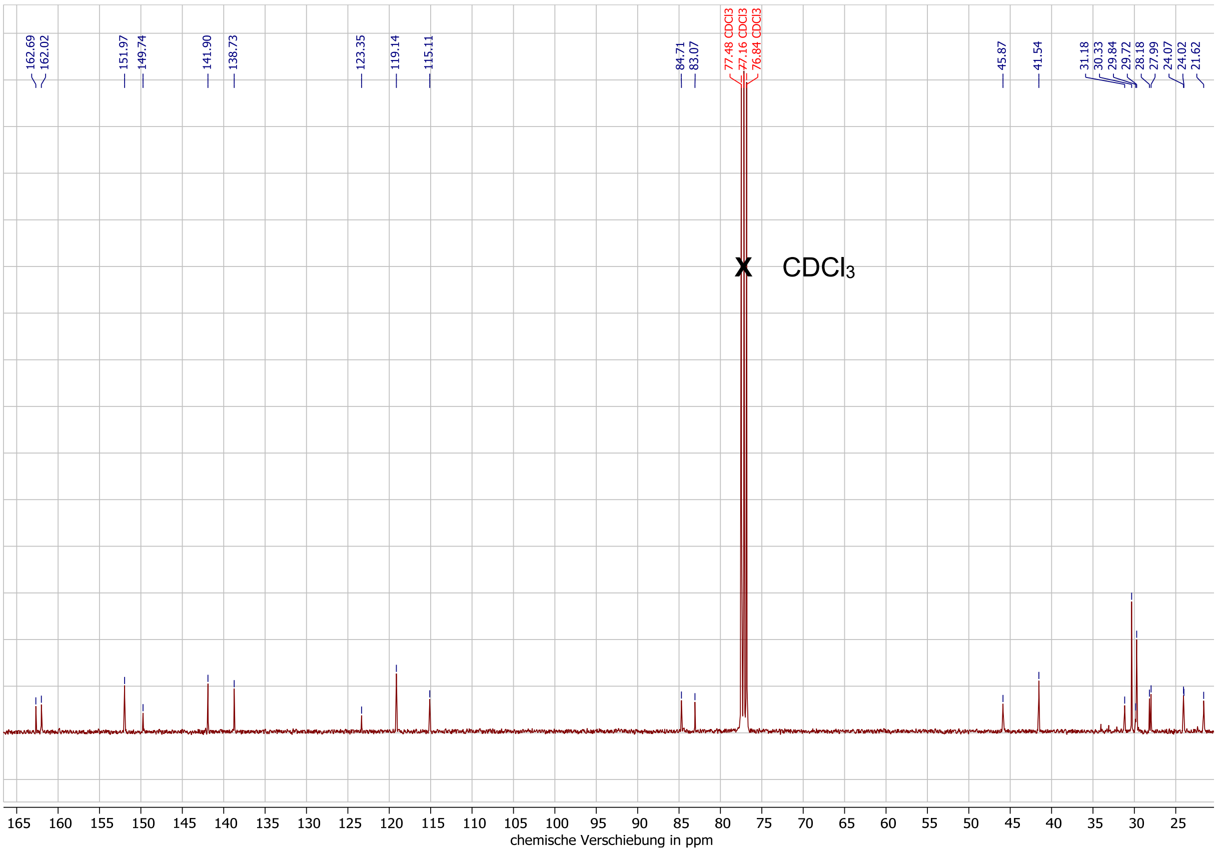


**LU14** $^{19}$F-NMR

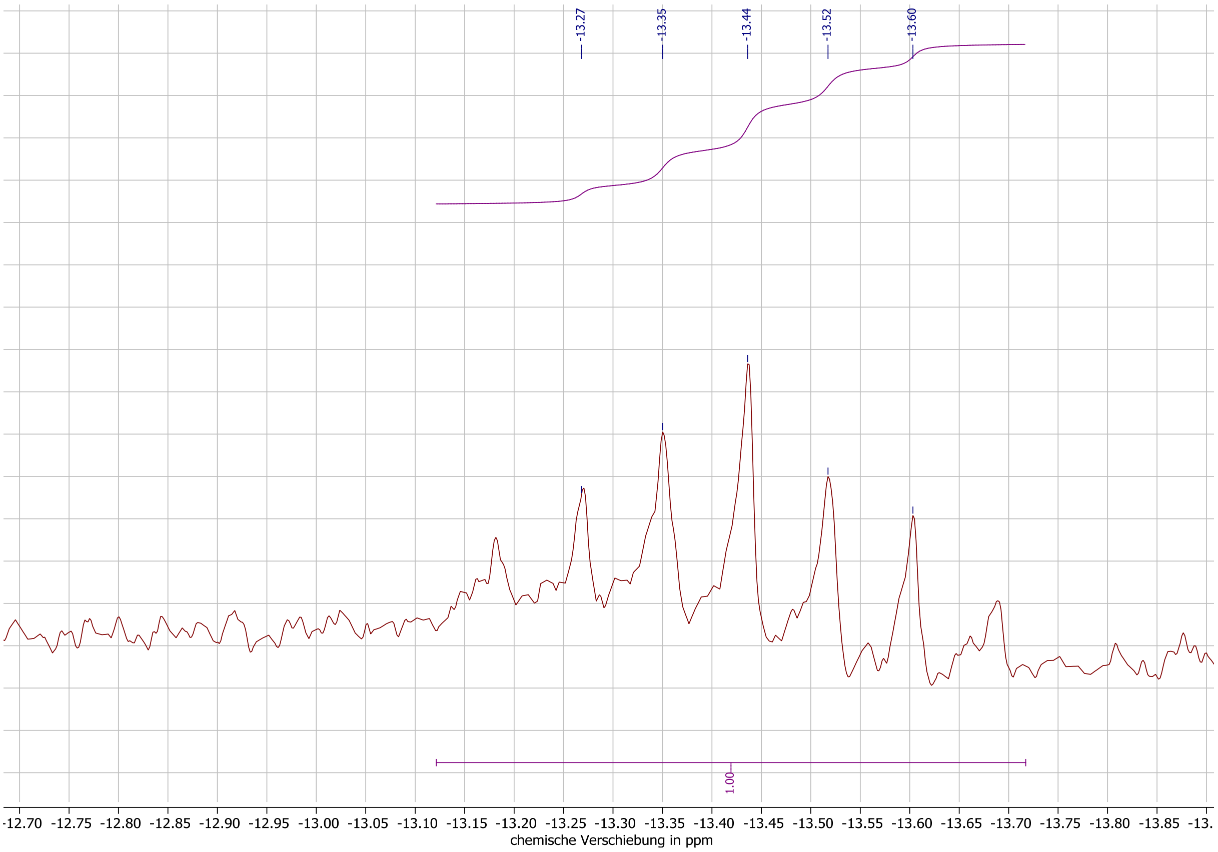

**LU14** APT:

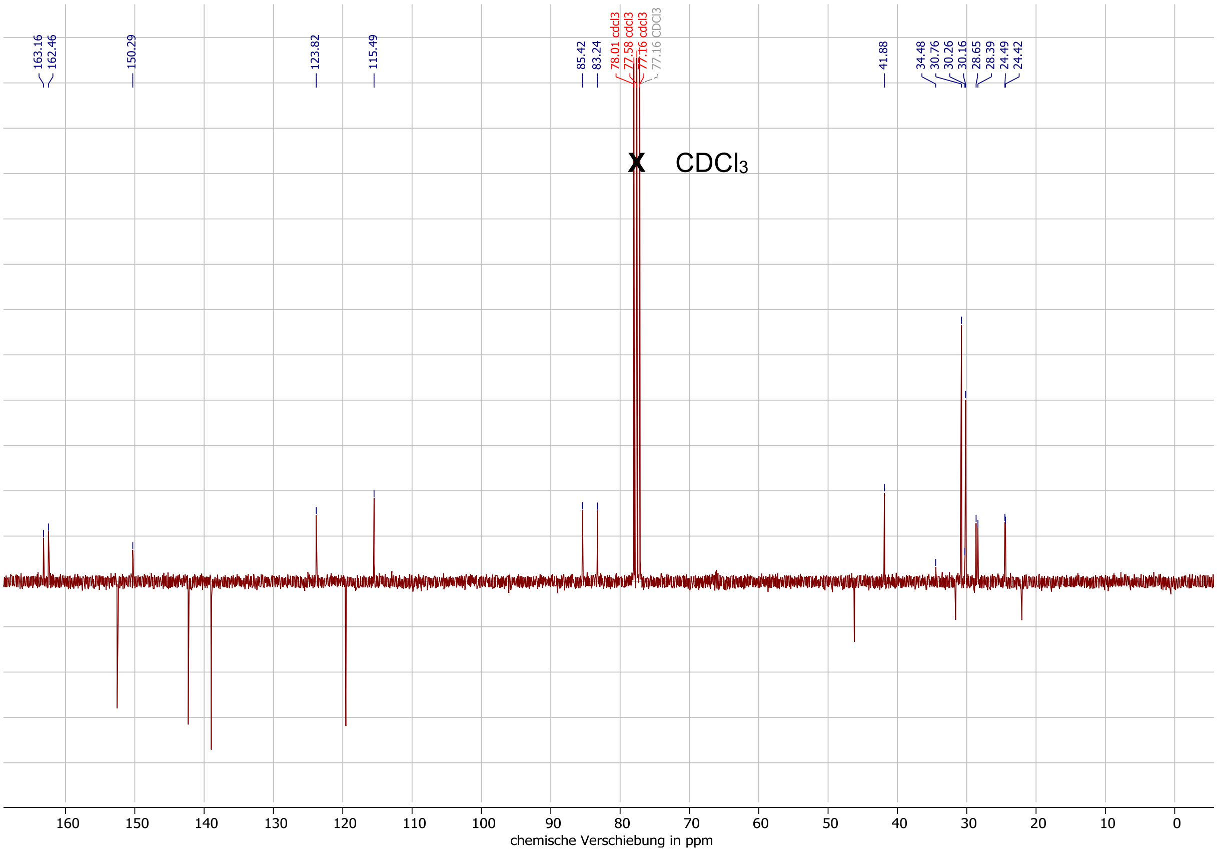


**LU14** 2D-NMR: COSY:

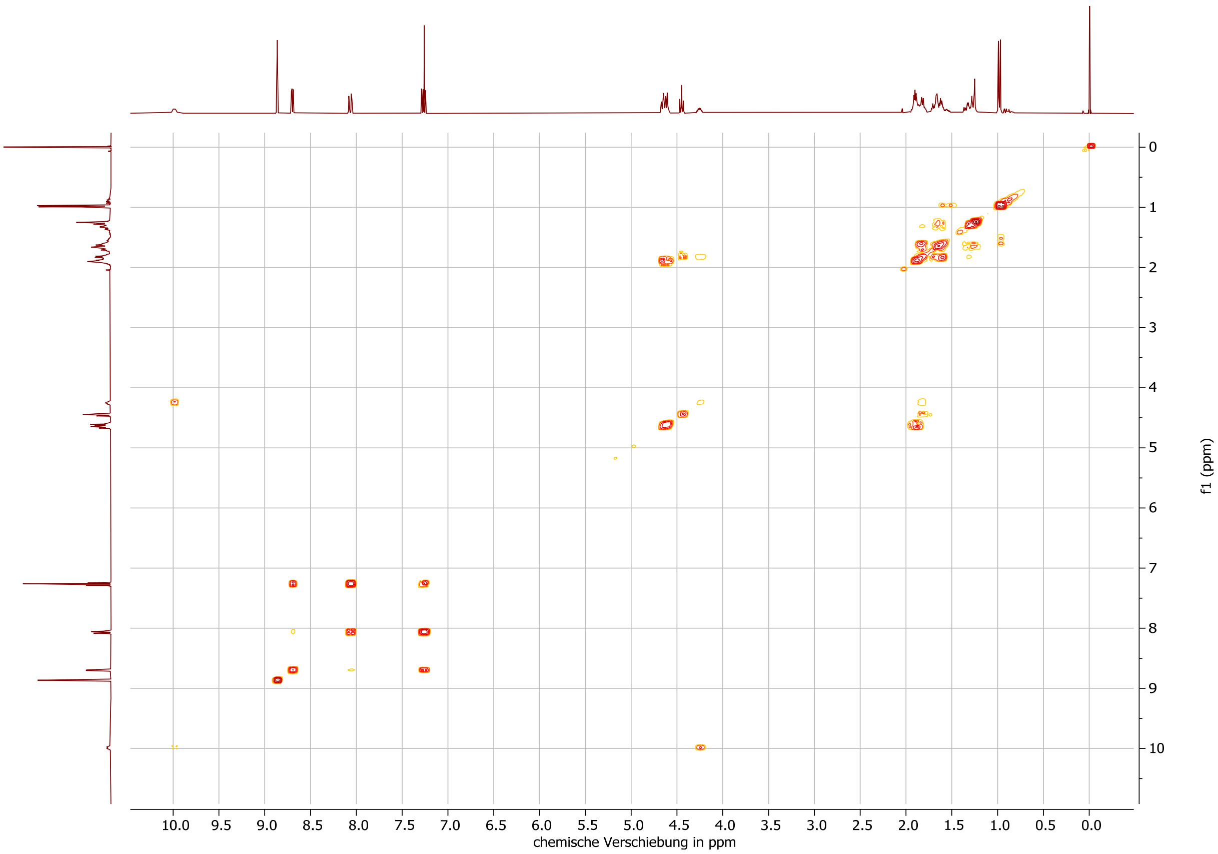

**LU14** 2D-NMR: NOESY:

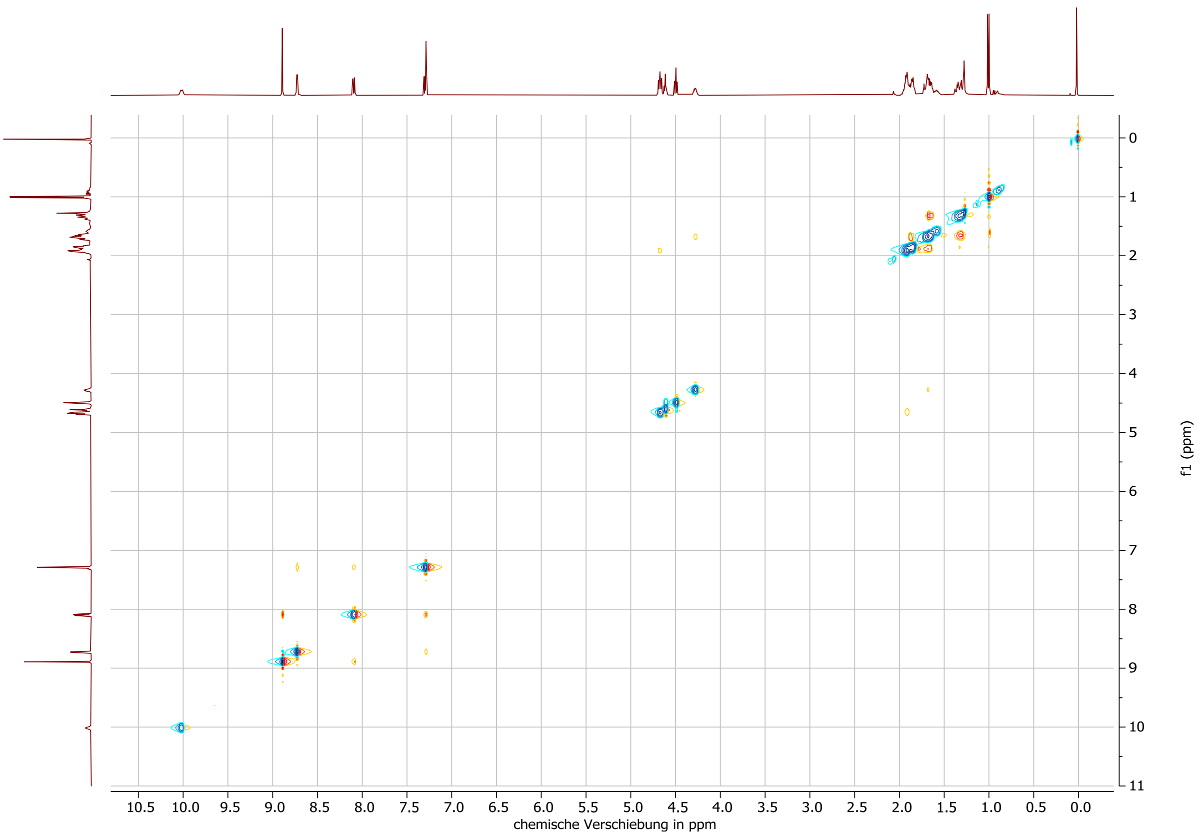


**LU14** 2D-NMR: HMBC:

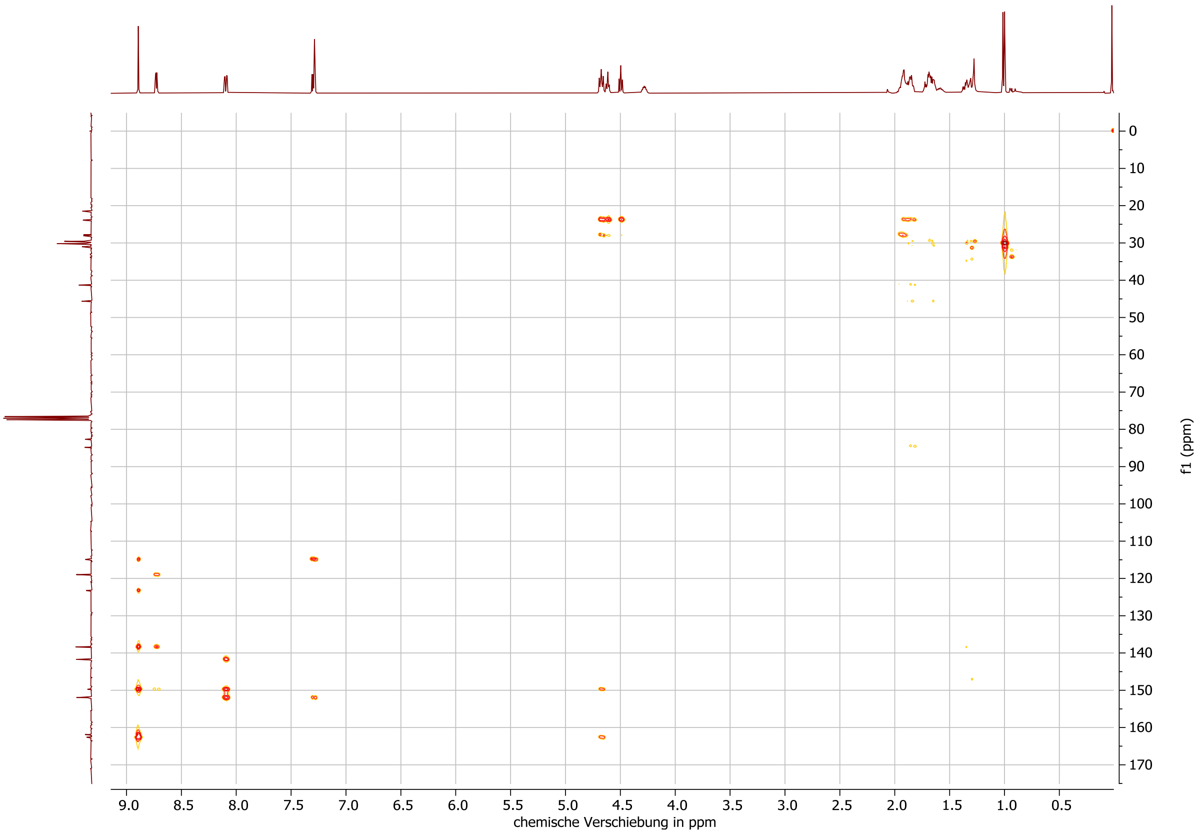

**LU14** 2D-NMR: HSQC:

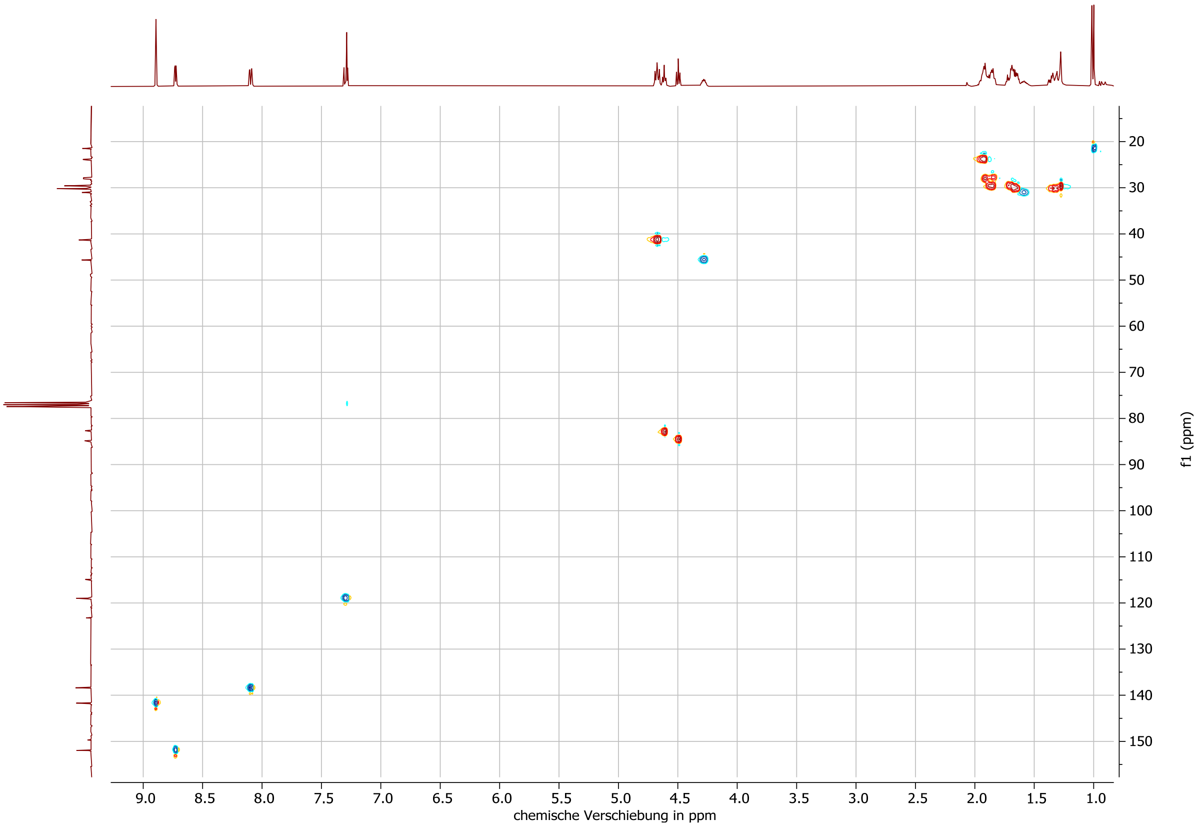


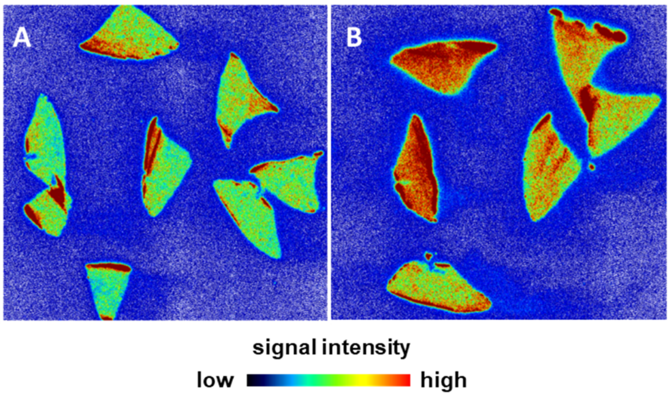


**Figure S1.** Radioluminography of rat spleen sections to analyze the distribution pattern [$^{18}$F]**LU14**. Cryosections (10 µm; female SPRD rats) were incubated with 1.3 nM [$^{18}$F]**LU14** without (total binding; (**A**) or with 5 µM GW405833 (nonspecific binding; (**B**) for 60 min.

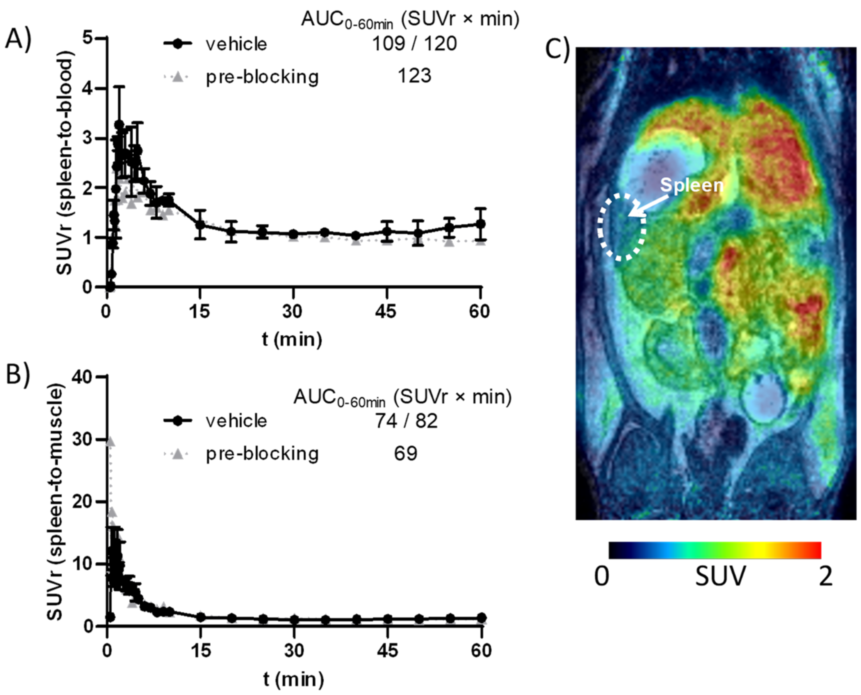


**Figure S2.** Dynamic PET imaging of [$^{18}$F]**LU14** in Wistar rats. Time-activity curves (mean ± S.D.) of SUV ratios of spleen-to-blood (**A**) and spleen-to-muscle (**B**) with (*n* = 2) and without (*n* = 1) pre-blocking with 1.5 mg/kg bodyweight applied i.v. 10 min prior tracer; (**C**) coronal plane, averaged time frames from 0–60 min.
